# NR4A3 inhibits the tumor progression of hepatocellular carcinoma by inducing cell cycle G0/G1 phase arrest and upregulation of CDKN2AIP expression

**DOI:** 10.7150/ijbs.95174

**Published:** 2024-10-28

**Authors:** Xinge Zhao, Xuejie Min, Zhenyu Wang, Xiaoxia Chen, Chao Ge, Fangyu Zhao, Hua Tian, Taoyang Chen, Jinjun Li

**Affiliations:** 1 State Key Laboratory of Systems Medicine for Cancer, Shanghai Cancer Institute, Renji Hospital, Shanghai Jiao Tong University School of Medicine, Shanghai 200032, China.; 2 Department of Oncology, Shanghai General Hospital, Shanghai Jiaotong University School of Medicine, Shanghai 200080, China.; 3 School of Life Science and Technology, Shanghai Tech University, Shanghai 201210, China.; 4 Department of Pathology, Qi Dong Liver Cancer Institute, Qidong 226220, China.

**Keywords:** hepatocellular carcinoma, NR4A3, CDKN2AIP, cell cycle, tumor progression

## Abstract

Nuclear receptor subfamily 4 group A member 3 (NR4A3) is a member of the orphan nuclear receptor superfamily, and exhibits transcription factor activity by binding to sequence-specific DNA. Considering that the specific mechanism by which NR4A3 regulates gene transcription in HCC (hepatocellular carcinoma) has not yet been elucidated, our study aimed to explore the transcriptional role of NR4A3 in regulating the target gene CDKN2AIP (CDKN2A interacting protein), which will suppress the development of HCC. Our data show that NR4A3 is downregulated in human HCC tissues, and that low expression of NR4A3 is correlated with poor prognosis, indicating that NR4A3 could act as a tumor suppressor gene in HCC. NR4A3 overexpression suppresses cell proliferation, clone formation, cell cycle arrest at G0/G1 phase and tumor growth *in vitro* and *in vivo* and promote DNA damage. NR4A3 could directly regulate the expression of CDKN2AIP at the transcriptional level, suggesting that NR4A3 may play a role as a transcription factor in HCC and may serve as a potential biomarker for predicting prognosis for HCC patients.

## Introduction

Liver cancer is one of the most common malignancies worldwide, and is one of the leading causes of death from cancer 1. The latest statistics showed that between 2015 and 2019, the incidence of liver cancer leveled off, with the progression of liver cancer mostly confined to men, with the incidence of liver cancer declining by about 2.6% per year in men under 50 and remaining unchanged in men over 50, while among women, the incidence of liver cancer in both age groups is increasing at a rate of 1.6-1.7% per year 2. On the other hand, current survival for liver cancer is only 21%, of which is not optimistic. The fact that there are natural genetic determinants of liver cancer cannot be ignored. As is the case with most other cancers, the expression of a patient's own genes reflects and is reflected in a number of factors, including the course of liver cancer, the degree of associated morbidity, and survival 3, 4.

Hepatocellular carcinoma (HCC) accounts for approximately 90% of primary liver cancers, and is a complex process caused mainly by infection with the hepatitis B virus (HBV) or hepatitis C virus (HCV), obesity, alcohol consumption, etc. 5. HCC is recognized as the fourth leading cause of cancer-related death globally, with the World Health Organization projecting that more than 1 million people will die from liver cancer each year by 2030 6, 7. Although much is known about HCC and there are many improvements in HCC treatment, the poor prognosis of HCC remains an urgent problem to solve 8. Therefore, it is necessary to clarify the pathogenesis of HCC to identify effective therapeutic strategies.

Transcription factors (TFs), as the components of regulating expression of genes, play important roles in tumorigenesis 9-11. Nuclear receptor subfamily 4 group A member 3 (NR4A3), also known as neuron-derived orphan receptor 1 (NOR1), belongs to the NR4A family, which includes orphaned receptors with no known endogenous ligand 12, 13. NR4A3 is a transcriptional activator that can efficiently bind to the NGFI-B response element-AAAGGTCA (NBRE) motif 14. Previous studies have demonstrated that NR4A3 is widely expressed in different types of tissues, such as the skeletal muscle, adipose tissue, T cells, liver, and brain 15. It can be induced by a wide range of physiological signals, such as fatty acids, stress, prostaglandins, growth factors, calcium, inflammatory cytokines, peptide hormones, and neurotransmitters, and most studies on NR4A3 have focused on immunity 16, 17. Although NR4A3 plays an important role in maintaining cellular homeostasis and pathophysiology, it is reportedly dysregulated in multiple cancer types, and has paradoxical roles in tumorigenesis 18, 19. NR4A3 activates proliferation of quiescent hepatocytes, and is required for hepatocyte proliferation after partial hepatectomy in mice, and is upregulated in human HCC samples, which was in contrast to our view 20. Interestingly, a study published in 2020 reported that NR4A3 is a target of LINC00467, and is downregulated in HCC 21. However, the mechanism by which NR4A3 inhibits the progression of HCC is yet to be elucidated.

This study found that CDKN2AIP (CDKN2A interacting protein) was a direct target of NR4A3. CDKN2AIP (CARF) was first identified as a novel ARF-binding (alternative reading frame) protein that stabilizes p53-tumor suppressor protein in an ARF-dependent/-independent manner 22. Analysis of the CARF promoter sequence provided clues on its function in several unique mechanisms, including stress sensing, gene regulation, and oncogenic responses 23.

In the present study, we revealed that NR4A3 acts as a tumor suppressor gene in human primary HCC, and that low expression of NR4A3 predicts poor prognosis in HCC patients. The results of our *in vitro* and *in vivo* studies showed that NR4A3 overexpression inhibited HCC cell proliferation, tumor formation and promote DNA damage. The results of flow cytometry analysis revealed that NR4A3 might affect the regulation of G0/G1 phase arrest of the cell cycle. We then conducted a series of studies to explore the mechanisms by which NR4A3 inhibits HCC progression and we found that NR4A3 functions by regulating the transcriptional activity of CDKN2AIP in HCC. In summary, we demonstrate that NR4A3 affects cell cycle G0/G1 phase arrest by regulating CDKN2AIP and ultimately regulating HCC progression. This is the first study to clarify that CDKN2AIP is a direct binding target of NR4A3 in HCC cells.

## Results

### NR4A3 expression is down-regulated and associated with prognosis of HCC patients

**Figure 1:**
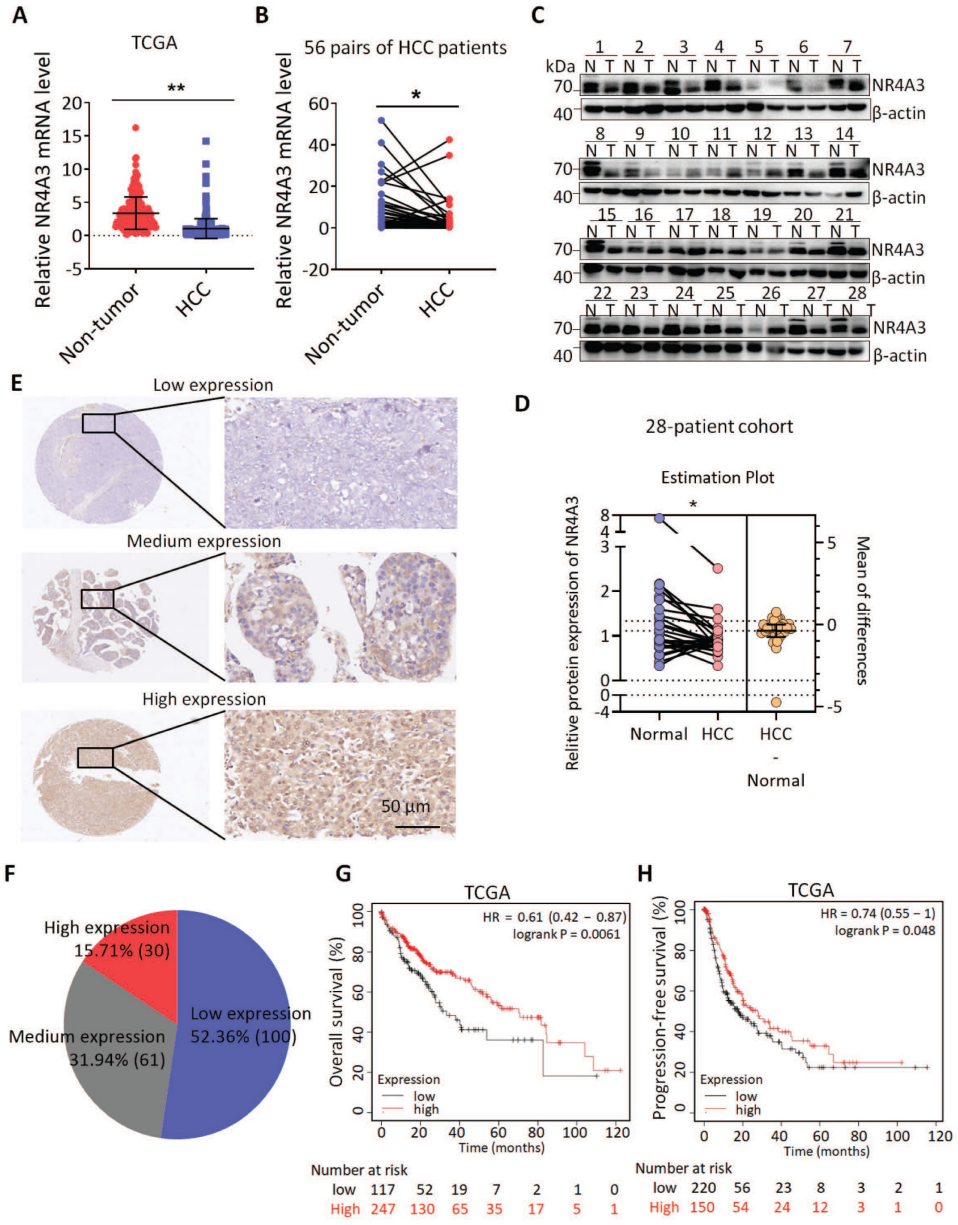
** NR4A3 expression is down-regulated and associated with prognosis of HCC patients.** (A) NR4A3 expression in HCC tissues and the corresponding normal tissues in The Cancer Genome Atlas (TCGA) datasets (log2 TPM, two-sided unpaired t-test). (B) NR4A3 mRNA expression in 56 paired HCC tissues and the adjacent matched noncancerous tissues were determined using qPCR. For qPCR, values were normalized with GAPDH. (C) The protein levels of NR4A3 in 28 paired HCC (T) and adjacent normal (N) samples were measured using western blot. β-actin was used as a loading control. (D) NR4A3 protein expression in 28 paired HCC tissues and the adjacent matched noncancerous tissues were quantified and analyzed. (E-F) The expression of NR4A3 in HCC tissues was cored, and representative IHC images showed the expression of NR4A3 in HCC tissues. (G-H) The correlation between NR4A3 expression and overall survival (G, n=364) and progression-free survival (H, n=370) of HCC patients in TCGA dataset was assessed by Kaplan-Meier survival analysis. The data were presented as mean ± SD. *p<0.05; **p<0.01.

To explore the role of NR4A3 in HCC, we first investigated the expression of NR4A3 using datasets from The Cancer Genome Atlas (TCGA). The results indicated that the expression of NR4A3 was downregulated in HCC tissues compared with that in noncancerous tissues (Fig. 1A and Fig. S1B). TCGA dataset showed that HCC was one of the tumors displaying significant downregulation of NR4A3 (Fig. S1A). We then analyzed NR4A3 mRNA expression in 56 pairs of human primary HCC tissues and matched adjacent noncancerous liver tissues using quantitative (q) RT-PCR and found that NR4A3 mRNA expression was frequently downregulated in HCC tissues compared with matched adjacent noncancerous liver tissues (Fig. 1B). To confirm these findings further, western blot was performed, and the results showed that the protein level of NR4A3 was markedly lower in HCC tissues (Fig. 1C-1D, Fig. S1D). Furthermore, we analyzed NR4A3 protein expression in paired HCC patients using immunohistochemistry (IHC) (Fig. 1E). We detected the expression of NR4A3 in 191 HCC cases using three groups: low, medium, and high NR4A3 expression. Of the 191 pairs, 100 (52.36%) had lower NR4A3 protein expression in tumor liver tissues than in noncancerous tissues, 61 (31.94%) had similar levels, and only 30 (15.71%) had higher NR4A3 protein expression (Fig. 1F). These results suggested that NR4A3 is downregulated in HCC tissues.

According to the group situation of IHC, protein expression of NR4A3 was closely associated with gender and tumor size (Table 1). The expression of NR4A3 was negatively associated with tumor stage in TCGA dataset (Fig. S1C). In addition, Kaplan-Meier survival analysis showed that low NR4A3 expression levels were correlated with poor prognosis of overall survival (OS) and progression-free survival (PFS) of HCC patients in the TCGA cohort (Fig. 1G-1H). These findings indicate that NR4A3 may play an important role in HCC, and that low expression of NR4A3 is associated with the malignant progression of HCC.

### Overexpression of NR4A3 inhibits HCC cell proliferation and tumorigenicity

**Figure 2:**
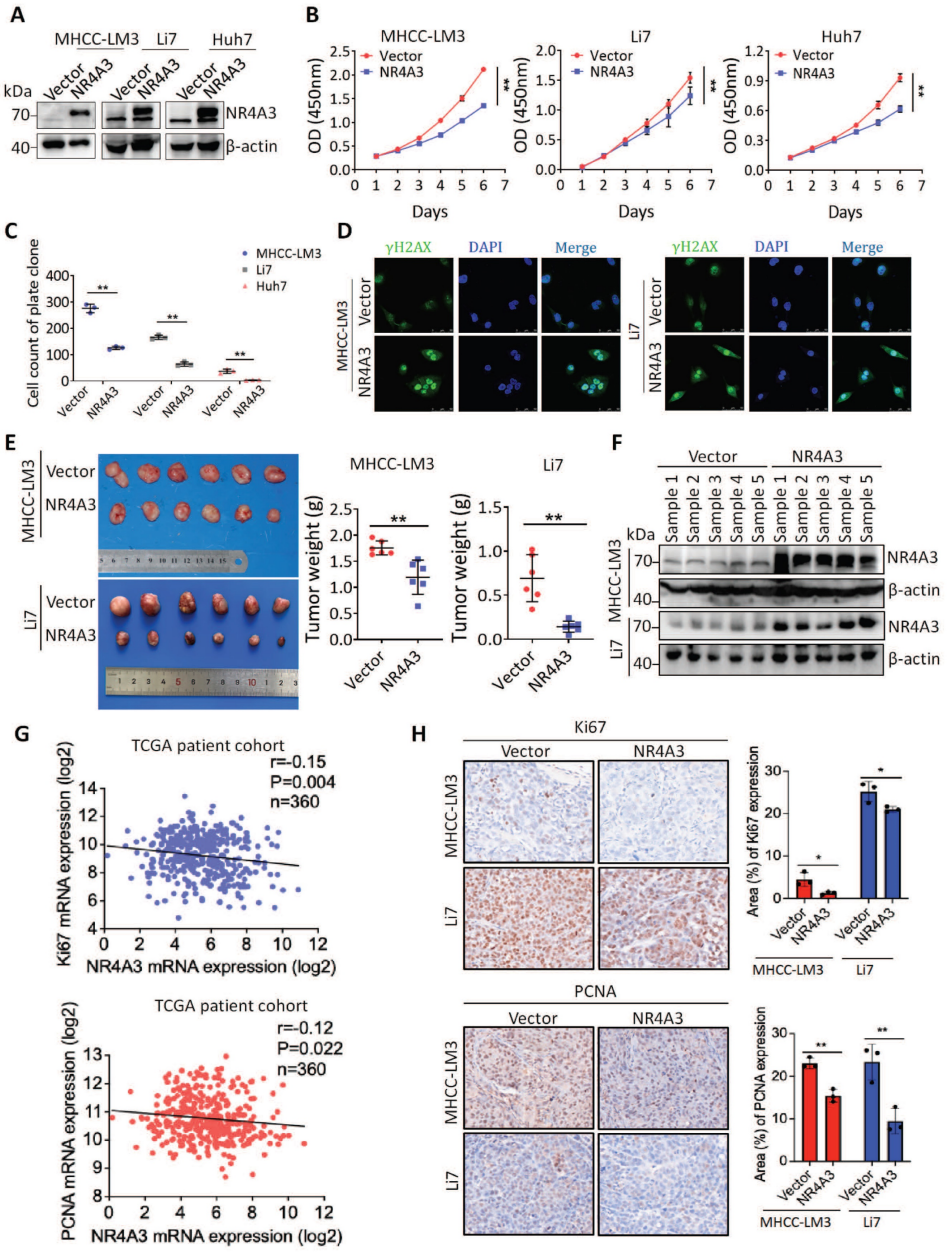
** Overexpression of NR4A3 inhibits HCC cell proliferation and tumorigenicity.** (A) Western blot showed that NR4A3 was efficiently up-regulated in MHCC-LM3, Li7, and Huh7 cells. (B) CCK8 assay revealed that the proliferation of HCC cells was inhibited by overexpression of NR4A3. (C) Colony formation assay showed that overexpression of NR4A3 inhibited the proliferation of HCC cells. The bar graph showed quantitative analysis data with three replicates. (D) Immunofluorescence showed overexpression of NR4A3 enhanced γ-H2AX foci. (E) *In vivo* growth assays showed the difference in tumor weight between NR4A3 overexpressed cells and the vector group in MHCC-LM3 (top panel) and Li7 (bottom panel) cell lines. (F) Western blot analysis showed that the expression of NR4A3 was significantly up-regulated in NR4A3-overexpressing tumor tissues collected from nude mice with tumor xenografts. β-actin was used as a loading control. (G) The correlation between NR4A3 and Ki67 or PCNA mRNA expression in HCC tissues was analyzed in The Cancer Genome Atlas (TCGA) dataset. (H) Immunohistochemical images of Ki67 and PCNA expressions in xenograft tumors derived from MHCC-LM3 and Li7 cells with NR4A3 overexpression. Original magnification: × 400. The positively stain area (in percentages) were analyzed. *, p<0.05; **, p<0.01.

Expression of NR4A3 is associated with tumor size in HCC, hence, we speculated that NR4A3 may be important for HCC tumor growth. To confirm this possibility, MHCC-LM3, Li7, and Huh7 cells were chosen for upregulation studies owing to their low endogenous NR4A3 expression in HCC cell lines (Fig. S2A-S2B). The CCK-8 and plate colony formation assays for analyzing cell viability indicated that overexpression of NR4A3 inhibited HCC cell proliferation (Fig. 2A-2C, Fig. S2C and S3A). To determine the effect of NR4A3 on DNA damage, we examined the γ-H2AX foci by immunofluorescence, and the results showed that overexpression of NR4A3 promoted γ-H2AX formation (Fig. 2D).

We then applied stable NR4A3 overexpressing MHCC-LM3 or Li7 cells to determine whether NR4A3 could inhibit tumorigenesis in a xenograft model* in vivo*. Cells transfected with the empty vector were used as the control group. Tumors were separated from the mice after four weeks. Our results indicated that the average weight of the tumors was significantly decreased in NR4A3-overexpressing mice (Fig. 2E). Meanwhile, the tissues of xenografts overexpressing NR4A3 maintained high expression levels of NR4A3 (Fig. 2F and Fig.S2E-S2F). The expression of NR4A3 was negatively correlated with Ki67 and PCNA in TCGA dataset as well as in NR4A3-overexpressing tissues collected from nude mice with tumor xenografts (Fig. 2G). Moreover, the Ki67 and PCNA percentage area of tumor cells was relatively decreased in NR4A3-overexpressing group when compared with the vector control group (Fig. 2H). Taken together, these findings suggest that NR4A3 is a negative regulator of HCC proliferation *in vivo* and *in vitro*.

### Knockdown of NR4A3 promotes HCC cell proliferation and tumorigenicity

**Figure 3:**
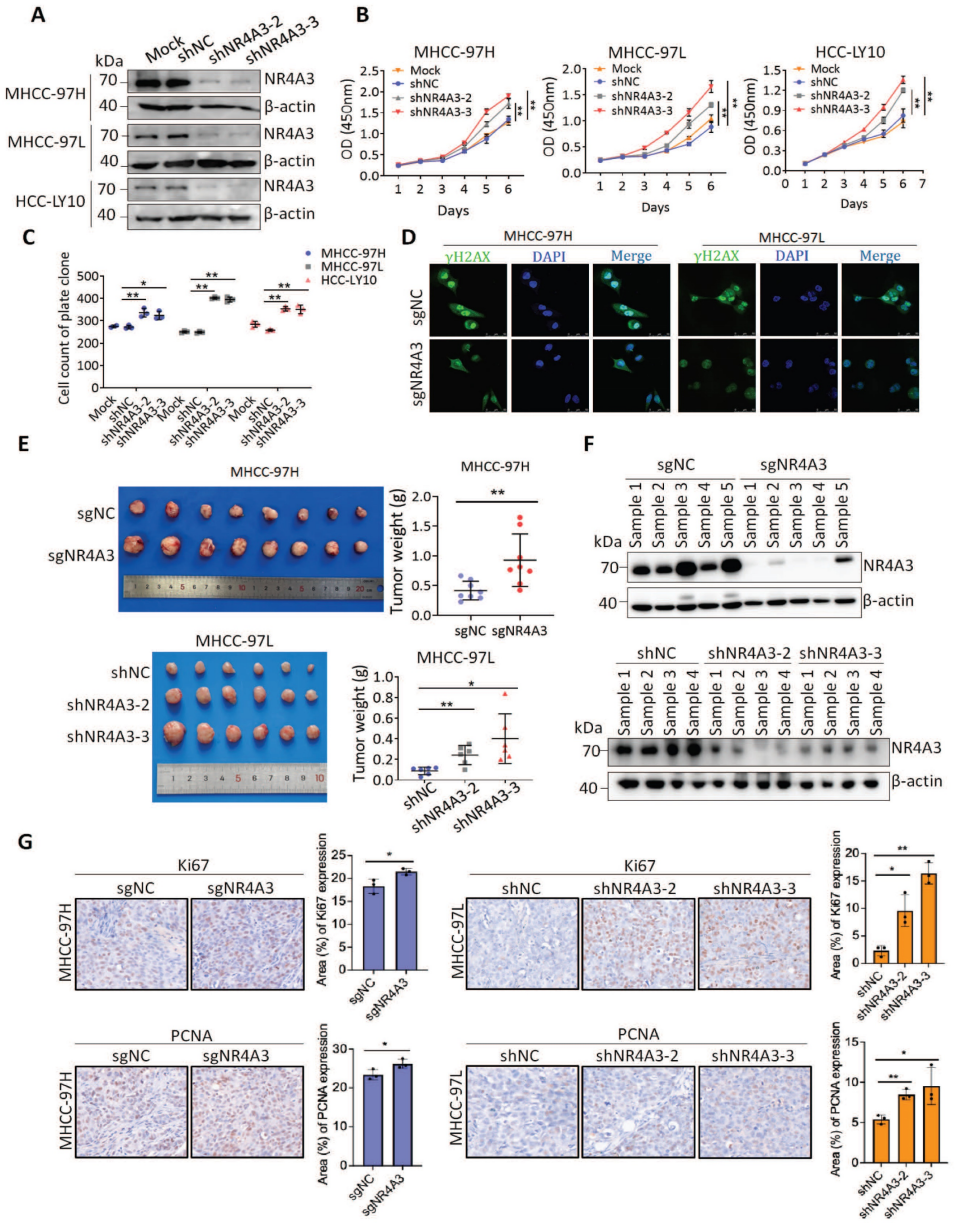
** Knockdown of NR4A3 promotes HCC cell proliferation and tumorigenicity.** (A) Western blot analysis of NR4A3 protein in MHCC-97H, MHCC-97L and HCC-LY10 cells stably transfected with shRNA or shNC. (B) CCK8 assay revealed that the proliferation of HCC cells was promoted by down-regulation of NR4A3. (C) Colony formation assay showed that down-regulation of NR4A3 promoted the proliferation of HCC cells. The bar graph showed quantitative analysis data with three replicates. (D) Immunofluorescence showed knockout of NR4A3 decreased γ-H2AX foci. (E) *In vivo* growth assays showed the difference in tumor weight between sgNC and sgNR4A3 group in MHCC-97H (top panel) and between shNC and shNR4A3 groups in MHCC-97L (bottom panel) cell lines. (F) Western blot analysis showed that the expression of NR4A3 was obviously down-regulated in NR4A3-shRNA tumor tissues collected from nude mice with tumor xenografts. β-actin was used as a loading control. (G) Immunohistochemical images of Ki67 and PCNA expressions in xenograft tumors derived from MHCC-97H and MHCC-97L cells with NR4A3 knockout or knockdown. Original magnification: × 400. The positively stain area (in percentages) were analyzed. *, p<0.05; **, p<0.01.

To determine the functional role of endogenous NR4A3, two lentiviral vectors expressing shRNAs targeting NR4A3 were used to knock down the expression of endogenous NR4A3 and CRISPR/Cas9 technology was used to contribute the knockout NR4A3 cell lines. We selected MHCC-97H, MHCC-97L, and HCC-LY10 cells to knock down NR4A3 and successfully established stably downregulated HCC cell lines (Fig. 3A and Fig. S2D) as well as NR4A3 knockout cell lines in MHCC-97H and MHCC-97L cells. As expected, knockdown of NR4A3 in MHCC-97H, MHCC-97L, and HCC-LY10 cells markedly promoted cell growth and colony formation *in vitro* (Fig. 3B-3C and Fig. S3B). Similarly, knockout of NR4A3 significantly enhanced cell proliferation by CCK-8 assay and clone formation assay (Fig. S3C-G). These findings validate the results obtained by the overexpression of NR4A3. Then we examined the γ-H2AX foci in NR4A3 knockout MHCC-97H and MHCC-97L cells, and the results showed that knockout of NR4A3 decrease γ-H2AX formation (Fig. 3D).

We then used stable NR4A3 knockout MHCC-97H cells and NR4A3 knockdown MHCC-97L cells to determine whether NR4A3 knockout or knockdown promotes tumorigenesis in a xenograft model* in vivo*. The cells transfected with sgNC or shNC served as the control group. Tumors were separated from the mice after four weeks. Our results indicated that the average weight of tumors increased in NR4A3-knockout or knockdown mice (Fig. 3E). Meanwhile, xenograft knockdown NR4A3 maintained a low expression level of NR4A3 (Fig. 3F and Fig.S2G-S2H). Additionally, the expression of NR4A3 was negatively correlated with Ki67 and PCNA in NR4A3 knockout or knockdown tumor tissues collected from nude mice with tumor xenografts. And the Ki67 and PCNA percentage area of tumor cells was relatively increased in NR4A3 knockout or knockdown group when compared with their control group (Fig. 2G). Taken together, these studies indicate that NR4A3 is a negative regulator of HCC cell proliferation *in vivo* and *in vitro*.

### NR4A3 induces cell cycle arrest at the G0/G1 to S phase

**Figure 4:**
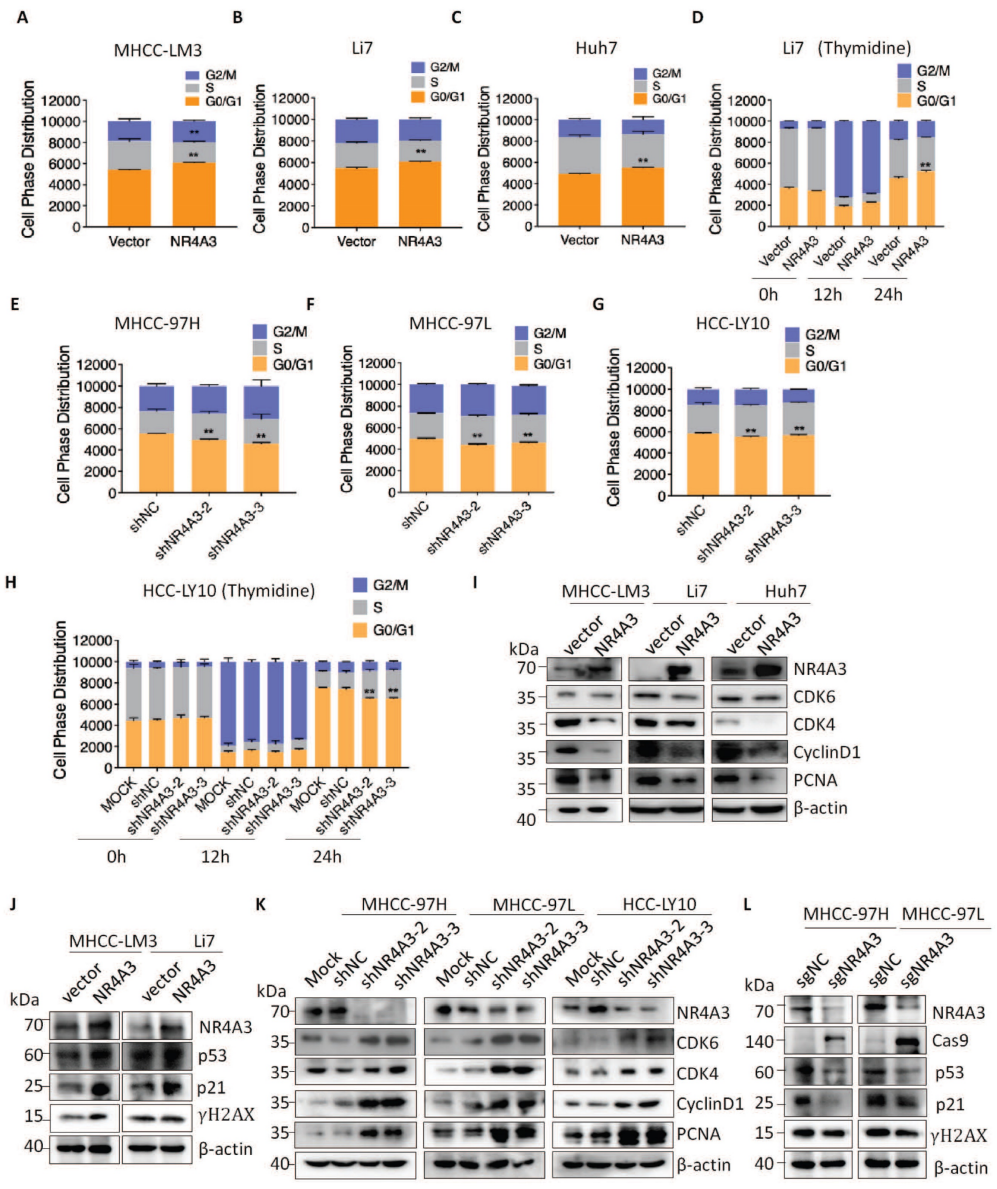
** NR4A3 induces cell cycle arrest at the G0/G1 to S phase.** The cell cycle distribution was showed in MHCC-LM3 (A), Li7 (B), and Huh7 (C) cells transfected with NR4A3 or the control vectors. (D) The cell cycle distribution of Li7 cells (vector/NR4A3) collected at 0, 12, and 24 h after synchronizing with 2 mM thymidine. The cell cycle distribution was showed in MHCC-97H (E), MHCC-97L (F) and HCC-LY10 (G) cells that were stably transfected with NR4A3-shRNAs or shNC vectors. (H) The cell cycle distribution of HCC-LY10 cells (Mock/shNC/shNR4A3-1/shNR4A3-2) collected at 0, 12, and 24 h after synchronizing with 2 mM thymidine. Western blot analysis of cell cycle associated proteins in the G1 phase (CDK6, CDK4, CyclinD1) and proliferation marker (PCNA) expression in NR4A3 overexpressing cells (I) and NR4A3 knockdown cells (K). (J) Western blot analysis showed that p53, p21 and γH2AX was efficiently up-regulated in NR4A3 up-regulated MHCC-LM3 and Li7 cells. (L) Western blot analysis showed that p53, p21 and γH2AX was efficiently decline in NR4A3 knockout MHCC-97H and MHCC-97L cells. β-actin was used as a loading control. *, p<0.05; **, p<0.01.

To further investigate the inhibitory effect of NR4A3 on HCC cell proliferation, the cell cycle distribution of stable NR4A3 overexpressing MHCC-LM3, Li7, and Huh7 cells was evaluated using flow cytometry (Fig. 4A-4C). The results showed that the percentage of cells in the G0/G1 phase was higher in the NR4A3 overexpression group than in the control group. We also used stable downregulated NR4A3 MHCC-97H, MHCC-97L, and HCC-LY10 cells and stable NR4A3 knockout MHCC-97H, MHCC-97L cells to examine cell cycle distribution (Fig. 4E-4G and Fig. S3H-I). After NR4A3 knockout or knockdown, the opposite effects were observed. Next, 2 mM thymidine was used to synchronize the cells at the G0/G1 phase border. Cells were collected at 0, 12, and 24 h. Flow cytometry analysis showed that the percentage of cells in the G0/G1 phase was significantly higher in NR4A3 overexpressing Li7 cells than in control cells after thymidine treatment (Fig. 4D). In addition, the opposite result was observed in NR4A3 down-regulated HCC-LY10 cells (Fig. 4H). Next, we detected the expression of essential molecules that regulate the G0/G1 to S phase transition in NR4A3-overexpressing and control cells. Upon NR4A3 overexpression or downregulation, the protein levels of p21, p53, CyclinD1, CDK4, CDK6, and PCNA as well as the DNA damage related γ-H2AX changed accordingly (Fig. 4I-4L and Fig. S3J). This implied that the overexpression of NR4A3 resulted in G0/G1 to S phase arrest and might promoted DNA damage in HCC cells.

### NR4A3 suppressed HCC progression by inducing CDKN2AIP expression

**Figure 5:**
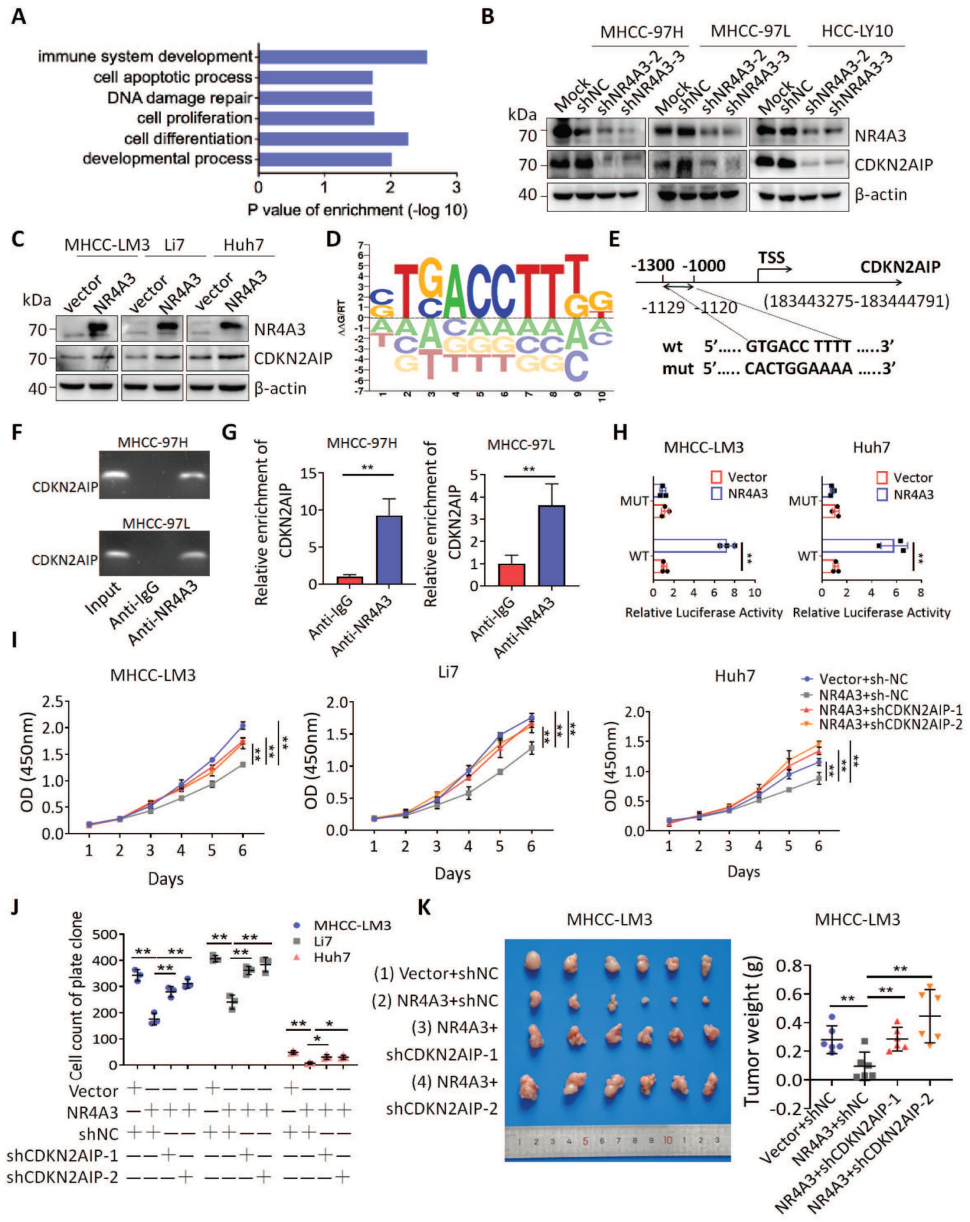
** NR4A3 suppressed HCC progression by inducing CDKN2AIP expression.** (A) The panel showed the overlap influenced signal pathway after over-expressing of NR4A3 in MHCC-LM3 cells. Western blot analysis of CDKN2AIP protein expressions examined in NR4A3 knockdown cells (B) and NR4A3 overexpressing cells (C). (D) Potential NR4A3-binding sites in the CDKN2AIP promoter identified with the database. (E) The panel is the sequence logo of NR4A3 potential binding site and mutant sites in the CDKN2AIP promoter. (F-G) Binding of NR4A3 to the CDKN2AIP promoter was performed by ChIP using the antibody against NR4A3 and negative control (IgG) in MHCC-97H and MHCC-97L cells. (H) Relative activities of the wide-type and mutant-type CDKN2AIP promoter after transfection of NR4A3 and vector into MHCC-LM3 and Huh7 cells analyzed using luciferase assay. Data are mean ± S.D. from experiments with three replicates. (I) Knockdown of CDKN2AIP reversed the inhibitory effect of NR4A3 on cell proliferation using CCK-8 assay. (J) Knockdown of CDKN2AIP reversed the inhibitory effect of NR4A3 on cell colony formation. (K) MHCC-LM3 cells stably overexpressing NR4A3 with knockdown of CDKN2AIP were injected into one flank of nude mice. Tumors were weighed. *, p<0.05; **, p<0.01.

To elucidate the key molecules participating in NR4A3-inhibiting proliferation and tumorigenesis of HCC, we examined the transcriptome of MHCC-LM3-vector and MHCC-LM3-NR4A3 cells using RNA-Seq analysis and mainly focused on the upregulated genes in MHCC-LM3-NR4A3 cells. Furthermore, we performed gene ontology (GO) enrichment analysis and found that these genes were correlated with cell biological processes, such as cell proliferation, DNA damage repair, and cell apoptosis process (Fig. 5A). Subsequently, we screened a set of HCC-related genes and examined the mRNA levels of these genes in NR4A3-overexpressed MHCC-LM3 cells to validate the RNA-seq results. RT-qPCR verified that upregulation of NR4A3 significantly increased CDKN2AIP and VTN (vitronectin) levels in MHCC-LM3 (expression ratio showing greater than 2.0-fold difference compared with the control group) (Fig. S4A). Given the critical role of CDKN2AIP in cell proliferation progression 23, 24, we investigated the role and underlying mechanism of CDKN2AIP in the tumor inhibitory role of NR4A3 in HCC. RT-qPCR and western blotting were then performed to examine CDKN2AIP levels in HCC cells with NR4A3 overexpression or knockdown (Fig. 5B-C and Fig. S4B-S4G). The results further confirmed that CDKN2AIP is a potential downstream target gene of NR4A3.

Given these results and the fact that NR4A3 is a transcription factor, we speculated whether CDKN2AIP is a direct target of NR4A3. hTFtarget database was used to analyze the potential NR4A3-binding site in the CDKN2AIP promoter (Fig. 5D). Based on the sequence of the NR4A3 potential binding site on the hTFtarget website, we mutated the predicted binding site at the predicted NR4A3-binding site (-1129 to -1120 bp), as shown in Fig. 5E. The chromatin immunoprecipitation (ChIP) assay indicated that NR4A3-binding to the predicted sites in the CDKN2AIP promoter region (Fig. 5F-5G). And as Florence *et al.* have characterized Skp2 as a novel NOR1-regulated target gene and detail a previously unrecognized transcriptional cascade regulating mitogen-induced VSMC proliferation 25. We used Skp2 as a positive control for NR4A3 in the ChIP experiment to validate our results (Fig.S5A-S5B). Also, sgNR4A3 MHCC-97H and MHCC-97L cells were also used to confirm the specificity of antibody we used (Fig.S5C-S5F). The results of the luciferase assay further showed that the relative luciferase activity of the CDKN2AIP promoter was significantly induced by the overexpression of NR4A3, and the luciferase activity was reversed by transfection of the mutant (Fig. 5H). Taken together, CDKN2AIP promoter is a direct transcriptional target of NR4A3. These data suggested that NR4A3 promotes CDKN2AIP transcription by binding to its promoter.

CDKN2AIP overexpression causes growth arrest of human cancer cells and premature senescence of normal cells through activation of the p53 pathway 22, 26, we used the CCK-8 assay and plate colony formation assay to analyze cell viability. The results indicated that CDKN2AIP overexpression inhibited HCC cell proliferation (Fig. S4H-S4L). As CDKN2AIP has been reported to promote HCC progression 27, we investigated whether NR4A3 suppresses cell proliferation by upregulating CDKN2AIP. CCK-8 and clone formation assays revealed that the decrease in CDKN2AIP reversed NR4A3-induced inhibition of cell proliferation (Fig. 5I-5J and Fig. S6A and S6C-S6E). Meanwhile, the diminishment of tumor growth induced by NR4A3 overexpression were significantly inhibited by CDKN2AIP knockdown in MHCC-LM3 cells, which confirming the role of CDKN2AIP in NR4A3-mediated inhibition of cell proliferation (Fig.5K). The protein levels of NR4A3 and CDKN2AIP in mouse tumor tissues were examined by Western blot analyses (Fig. S6F). Therefore, the above data indicate that CDKN2AIP is a direct and functional target of NR4A3, and is responsible for the suppressive effects of NR4A3 in HCC cells.

### NR4A3 inhibits cell proliferation by up-regulating CDKN2AIP expression

**Figure 6:**
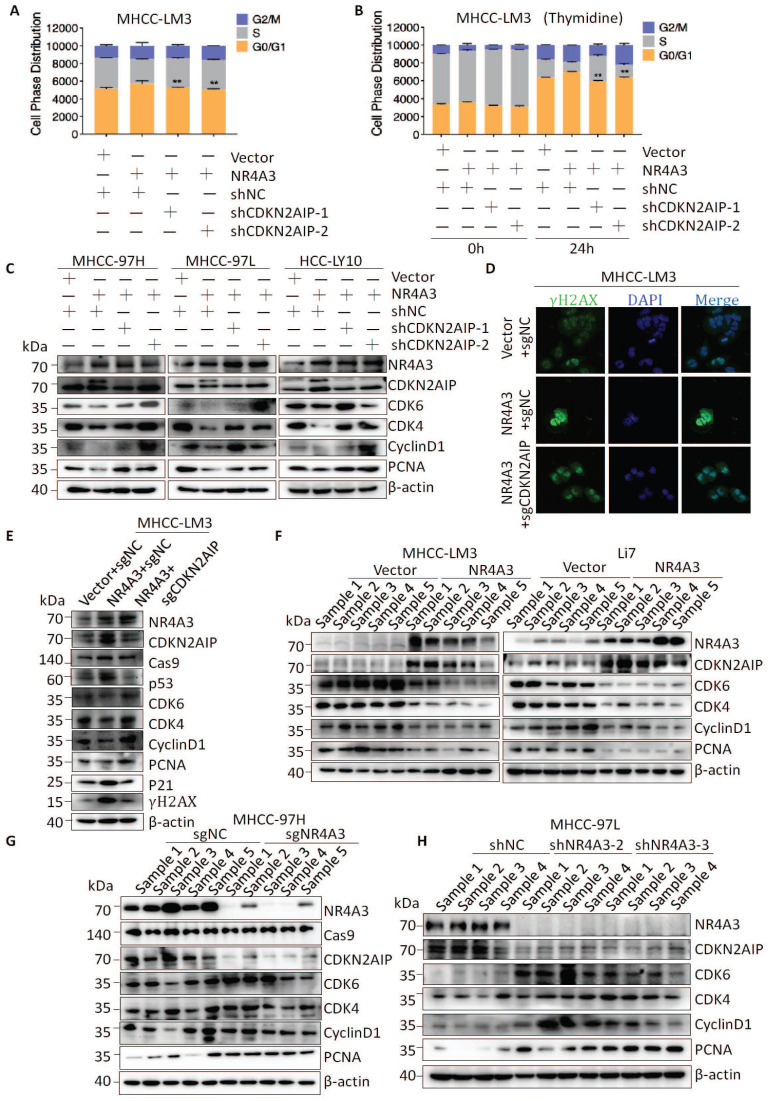
** NR4A3 inhibits cell proliferation by up-regulating CDKN2AIP expression.** (A) Knockdown of CDKN2AIP reversed the inhibitory effect of NR4A3 on cell cycle distribution was showed in MHCC-LM3 cells. (B) Knockdown of CDKN2AIP reversed the inhibitory effect of NR4A3 on cell cycle distribution collected at 0 and 24 h after synchronizing with 2 mM thymidine was showed in MHCC-LM3 cells. (C) Knockdown of CDKN2AIP reversed the inhibitory effect of NR4A3 on cell cycle-related proteins by western blot analysis. (D) Knockout of CDKN2AIP reversed γ-H2AX foci performed after overexpression of NR4A3 in MHCC-LM3 cells. (E) Knockout of CDKN2AIP reversed the protein expressions of cell cycle-related genes, PCNA and γH2AX examined in NR4A3-overexpressed MHCC-LM3 cells. Western blot analysis of cell cycle-related proteins in NR4A3-overexpressing tumor tissues (F) and in NR4A3-sgRNA (G)/shRNA tumor tissues (H) collected from nude mice with tumor xenografts. β-actin was used as a loading control. *, p<0.05; **, p<0.01.

To further clarify whether NR4A3 regulates the cell cycle by activating CDKN2AIP, we examined the cell cycle distribution and found that a decrease in CDKN2AIP reversed NR4A3-induced G1/S arrest of the cell cycle (Fig. 6A). Next, 2 mM thymidine was used to synchronize the cells at the G0/G1 phase border. Cells were collected at 0 and 24 h. Flow cytometry analysis showed that the decrease in CDKN2AIP reversed the NR4A3-induced increase in the percentage of cells in the G0/G1 phase in the MHCC-LM3 cells after thymidine treatment (Fig. 6B). We then probed the protein profile changes of CDKN2AIP, CyclinD1, CDK4, CDK6, and PCNA after knocking down CDKN2AIP under the condition of NR4A3 overexpression using western blot analysis (Fig. 6C). To verify the effect of NR4A3 on DNA damage whether mediated by CDKN2AIP, we examined the γ-H2AX foci and the results showed that knockout of CDKN2AIP reversed γ-H2AX foci formation induced by overexpression of NR4A3 (Fig. 6D). Next, we detected the expression of essential molecules that regulate the G0/G1 to S phase transition to determine whether they were reversed by knockout of CDKN2AIP. Upon knockout of CDKN2AIP, the protein levels of p21, p53, CyclinD1, CDK4, CDK6, and PCNA as well as the DNA damage related γ-H2AX reversed accordingly (Fig. 6E). This implied that the overexpression of NR4A3 resulted in G0/G1 to S phase arrest and might promoted DNA damage in HCC cells. In addition, similar protein expression was observed in NR4A3-overexpressed (Fig. 6F), NR4A3-knockout (Fig. 6G) and NR4A3-downregulated (Fig. 6H) mouse tumor xenografts. The results indicated that NR4A3 upregulated the protein expression of CDKN2AIP and inhibited the G0/G1 phase pathway activity in xenograft tumor tissues derived from NR4A3 overexpressed cells.

### Clinical correlation between CDKN2AIP and NR4A3

**Figure 7:**
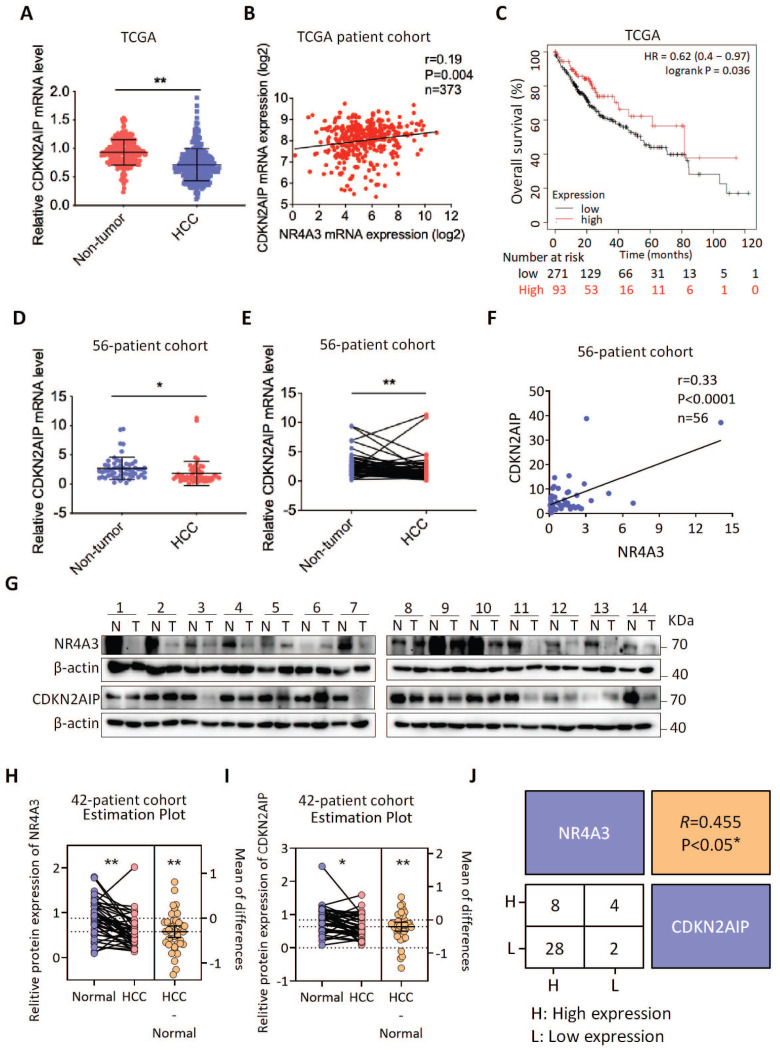
** Clinical correlation between CDKN2AIP and NR4A3.** The mRNA expression level of CDKN2AIP in HCC and their adjacent noncancerous liver tissues were analyzed in TCGA database (A) and 56 HCC patients (D-E). The correlation between the mRNA levels of CDKN2AIP and NR4A3 analyzed in TCGA database (B) and 56 HCC patients (F). (C) The correlation between CDKN2AIP expression and overall survival (n=364) of HCC patients in TCGA dataset was assessed using Kaplan-Meier survival analysis. (G) The protein levels of NR4A3 in 42 paired HCC (T) and adjacent normal (N) samples (1-14) were measured using western blot. β-actin was used as a loading control. NR4A3 (H) and CDKN2AIP (I) protein expression in 42 paired HCC tissues and the adjacent matched noncancerous tissues were quantified and analyzed. (J) Correlations among NR4A3 and CDKN2AIP protein levels in 42 paired HCC tissues and adjacent normal samples were examined by western blot. Number represents the number of tissue cases. H, higher expression; L, lower expression. *, p<0.05; **, p<0.01.

Given that NR4A3 increased CDKN2AIP expression in HCC cells, we analyzed the expression of NR4A3 and CDKN2AIP in HCC and matched adjacent noncancerous liver tissues in TCGA dataset and the HCC patients we collected. We found that the mRNA expression of CDKN2AIP was significantly downregulated in human HCC tissues in TCGA cohort compared with noncancerous HCC tissues (Fig. 7A). We then investigated the correlation between the expression levels of NR4A3 and CDKN2AIP in 373 human primary HCC tissues in TCGA cohort, and the results showed that there was a positive relationship between mRNA expression of NR4A3 and CDKN2AIP (Fig. 7B). Moreover, Kaplan-Meier survival analysis of TCGA database showed that low expression of CDKN2AIP indicated poor prognosis in HCC patients (Fig. 7C). We examined CDKN2AIP mRNA expression levels in 56 pairs of HCC tissues and matched adjacent noncancerous liver tissues. This result was consistent with the analysis in the TCGA cohort (Fig. 7D-7E). Notably, CDKN2AIP mRNA expression was positively associated with NR4A3 expression in HCC tissues (Fig. 7F). Moreover, western blot analyses were performed on 42 paired HCC and matched adjacent noncancerous liver tissues, and the results showed that CDKN2AIP was downregulated in HCC tissues compared with that in the paired adjacent liver tissues, and the levels of NR4A3 were lower in HCC tissues (Fig. 7G and Fig. S7A).

Statistical analysis showing the protein levels of NR4A3 (Fig. 7H) and CDKN2AIP (Fig. 7I) were both expressed lower in 42 paired HCC (T) samples compared with adjacent normal (N) samples. And protein levels of NR4A3 were positively correlated with the CDKN2AIP levels in 42 paired HCC tissues and the adjacent matched noncancerous tissues (Fig. 7J). Correlation analysis of NR4A3 and CDKN2AIP protein level from the western blot results which was in accordance with the analysis of mRNA levels in the TCGA cohort. Taken together, these results suggest that the expression levels of NR4A3 and CDKN2AIP are positively correlated in HCC tissues and both of them are promising prognostic indicators for HCC patients clinically.

## Discussion

In this study, our aim was to investigate the role of NR4A3 as a tumor suppressor in HCC. In recent years, studies have revealed the significant involvement of NR4A3 in tumorigenesis, particularly in hematological malignancies. Although data indicate that NR4A3 acts as an oncogene in AciCC (acinic cell carcinoma) and some other tumors, its tumor suppressor role is more evident in many malignancies. Considering that gene function varies across different cancers due to tissue heterogeneity and cellular contexts, we examined mRNA and protein expression levels of NR4A3 between HCC and adjacent noncancerous tissues. We found that NR4A3 was downregulated in HCC tissues. In addition, our IHC staining results suggest a close correlation between NR4A3 protein expression and gender as well as tumor size in HCC patients. Given the gender-specific disparity in liver cancer incidence as indicated by recent epidemiological data, it is plausible to hypothesize that the differential expression of NR4A3 between males and females may contribute significantly. Consequently, our subsequent investigation aims to elucidate whether the distinct NR4A3 expression in male liver cancer patients exerts an influence on their prognosis. Furthermore, low expression levels of NR4A3 were positively associated with poor prognosis among HCC patients based on TCGA dataset analysis. Therefore, we suggest that NR4A3 plays a crucial role as a tumor suppressor gene in HCC and could serve as a potential biomarker for predicting patients' prognosis.

NR4A3 belongs to the steroid-thyroid hormone retinoid receptor superfamily and functions as a transcriptional activator by efficiently binding to NGFI-B (nerve growth factor-induced gene B) response elements (NBRE) 28, 29. Our previous study showed that it acts as a tumor suppressor gene in breast cancer through regulation of the MEK (mitogen-activated protein kinase kinase) signaling pathway via miR-665 targeting 30. Additionally, p53 has been reported to target NR4A3 contributing to apoptosis induction observed within breast and lung tumors 31. Previously suggested roles for NR4A3 include acting as a tumor suppressor gene within human cancers such as hematologic neoplasms including acute myeloid leukemia 32, lymphoma or gastric cancer 33-35; however, it has also been reported to act an oncogene within AciCC or neuroblastoma 36-38 indicating its dual functionality across different cancers. What attracted us most is its downregulation within HCC where it functions primarily as a tumor suppressor, which is in accordance with our result 21. Furthermore, analysis of TCGA database also revealed significantly reduced NR4A3 expression in HCC tissues compared to normal liver tissues.

Through gain- and loss-of-function experiments both* in vitro* and *in vivo*, we demonstrated the tumor suppressor role of NR4A3 in hepatocellular tumorigenesis. Overexpression of NR4A3 inhibited HCC cell proliferation, cell cycle progression, clone formation and promoted DNA damage while subcutaneous neoplasia experiments showed that it suppressed tumor formation whereas knockout or knockdown promoted it. Additionally, overexpression of NR4A3 induced cell cycle arrest at the G0/G1 to S phase transition partly due to its growth inhibitory effect.

We also identified CDKN2AIP as a potential downstream target of NR4A3 through RNA-seq analysis. We found that NR4A3 directly binds to the promoter region of CDKN2AIP promoting transcriptional activation in HCC cells. The protein encoded by CDKN2AIP regulates DNA damage response via several signaling pathways including p53-HDM2-p21 (WAF1) pathway which is critical for DNA damage response 39. Our data not only showed that overexpression of NR4A3 upregulated CDKN2AIP resulting in cell cycle arrest at the G0/G1 to S phase transition, but also suggested that NR4A3 promoted p21, p53 and γH2AX expressed through the regulation of CDKN2AIP in HCC cells. And as expected, mRNA and protein levels were positively correlated with each other indicating that NR4A3 positively regulated CDKN2AIP and thereby inhibiting HCC progression. Furthermore, this study has identified other potential targets which could be further investigated.

CDKN2AIP, also known as CARF, may serve as a novel key regulator of the p53 pathway at multiple checkpoints 26. Despite limited research on CDKN2AIP, particularly beyond its relevance in DNA damage response 24, 40, 41, existing reports suggest that CDKN2AIP functions either as a tumor suppressor or an oncogene depending on the specific tumor type. Moreover, CDKN2AIP plays a crucial role in genome preservation and tumor suppression 41. Additionally, levels of CDKN2AIP during stress and post-stress conditions could potentially predict cell fate by influencing either cell death or enhanced proliferation and malignant transformation 42. Furthermore, CDKN2AIP acts as a transcriptional repressor of HDM2 to exert negative feedback on p53 through proteasome-mediated degradation 23. However, contrary to our perspective, previous studies have reported that CDKN2AIP acts as an oncogene and activates beta-catenin/TCF signaling in HCC 27. Recently conducted research identified significantly lower methylation rates in CDKN2AIP among the HCC and liver cirrhosis (LC) group compared to the LC only group 43. In this present study, we observed downregulation of both CDKN2AIP and NR4A3 in HCC tissues when compared with the adjacent normal liver tissues; furthermore, these two factors exhibited significant positive correlation with each other. *In vitro* experiments demonstrated that upregulation of CDKN2AIP markedly inhibited cell proliferation. Rescue experiments revealed that upregulated NR4A3 combined with downregulated CDKN2AIP promoted cell proliferation and reversed the progression of cell cycle arrest from G0/G1 to S phase by increasing levels of CDK4/CDK6/CyclinD1/PCNA and decreasing expression levels of p21/p53/γH2AX in HCC cells.

In conclusion, our findings provide initial evidence suggesting that NR4A3 suppresses HCC cell proliferation by regulating levels of CDKN2AIP expression, thereby playing a pivotal role as a tumor suppressor during HCC progression both *in vitro* and *in vivo* settings. Furthermore, we demonstrated that NR4A3 induces cell cycle arrest from G0/G1 to S phase and may promote DNA damage by decreasing levels of CDK4/CDK6/CyclinD1/PCNA and upregulating expression levels of p21/p53/γH2AX, thereby inhibiting HCC proliferation. Taken together, our results elucidates the role of NR4A3 in the pathobiology and clinical significance of HCC and the relevant molecular mechanism, which may help prevent pathogenesis and lead to the development of a potential biomarker for HCC. We propose that strategies to modulate NR4A3 expression could be a viable approach for therapeutic intervention. Since the nature of "tumor suppressor transcription factor" makes it seem like a difficult target for targeted therapies, an intuitive ideal to abrogate the inhibition of tumor suppressor genes. To treat HCC with low-expression of NR4A3, one approach we envision is to introduce NR4A3 protein or use gene therapy to deliver NR4A3 mRNA or DNA into HCC cells, thereby reinstating the expression of NR4A3 protein 44. In addition, investigating the molecules that negatively regulate NR4A3 and developing inhibitors against these regulators could indirectly lead to increased NR4A3 expression. It is worth noting that increasing the expression of NR4A3 may have different effects on cells, so the effects and potential risks need to be carefully evaluated and more studies are needed to support this.

## Materials and methods

### Human liver specimens and TCGA cohort

All of 191 of human primary hepatocellular carcinoma/matched adjacent noncancerous liver tissue samples were obtained from the Qidong Liver Cancer Institute (Qidong, China) and the First affiliated Hospital of Zhejiang University (Hangzhou, China). Informed consent was obtained from all patients who did not undergo any antitumor therapy prior to surgical resection, and the collection of tissue specimens for clinical analysis was approved by the University Ethical Committee. This study was approved by the Research Ethics Committee of Renji Hospital, Shanghai Jiao Tong University (Shanghai, China).

### The Cancer Genome Atlas

Data from 268 HCC patients and 243 noncancerous patients from The Cancer Genome Atlas (TCGA) database. Overall survival (OS) and progression-free survival (PFS) of HCC patients were analyzed using TCGA data from the Kaplan-Meier plotter database.

### Immunohistochemistry

IHC was performed as described in our previous article 45. Simply put, slices were incubated with NR4A3 overnight at 4°C and then evaluated blind by two independent observers. The antibodies used in this study were anti-NR4A3 (NBP2-46246, 1:50, Novus), anti-PCNA (13110S, 1:3000, CST), and anti-Ki67 (ab15580, 1:500, abcam). Scores of staining intensities were: 0, negative; 1, weak; 2, moderate; 3, strong. Scores of positively stained cell proportion were: 0, no positive; 1, <10%; 2, 10%-35%; 3, 35%-75%; 4, >75%. Then two independent pathologists scored the staining results. And that the score of 0-2 was considered low expression and the score of 3-4 was considered high expression.

### Cell lines and culture

Human HCC cell lines MHCC-LM3, MHCC-97L, and MHCC-97H were kindly provided by the Liver Cancer Institute of Zhongshan Hospital, Fudan University (Shanghai, China). The Huh7 cell line was obtained from Riken Cell Bank (Tokyo, Japan). The Li7 cell line was purchased from Shanghai BioLeaf Biotech Company Limited (Shanghai, China). The immortalized human normal hepatocyte cell lines, MIHA, were purchased from Cell Biology of the Chinese Academy of Sciences (Shanghai, China). An HCC-LY10 cell line was established in our laboratory. The HEK-293T cell line was obtained from American Type Culture Collection (ATCC) (Manassas, VA, USA). Cells were all cultured in Dulbecco's modified Eagle's medium (DMEM; Sigma-Aldrich) containing 10% fetal bovine serum (FBS; Gibco, New York, USA) and maintained at 37 ° C in a humidified atmosphere containing 5% CO_2_. All cell lines used in this study were free of mycoplasma contamination and authenticated by morphological observation.

### Quantitative real-time polymerase chain reaction (qRT-PCR)

Total RNA was extracted from human primary HCC tissue specimens and cell lines using TRIzol reagent (Invitrogen, CA, USA) and reverse-transcribed using a PrimeScriptTM RT Reagent kit (TaKaRa, Dalian, China). RT-qPCR was performed with a 7500 Real-Time PCR system (Thermo Scientific, MA, USA) using SYBR Green Master Mix, following the manufacturer's protocol (TaKaRa, Dalian, China). mRNA expression levels were normalized to those of GAPDH and quantified using the comparative CT (2^-ΔΔCT^) method. All primers used for RT-qPCR are listed in Table S1.

### Western blotting

Cells were harvested and lysed in RIPA buffer containing PMSF (100:1), protease inhibitor (100:1), and phosphatase inhibitor (1000:1) for 30 min on ice. The proteins were extracted by centrifugation at 12,000 g, 4 °C for 20 min. Protein in the supernatant was measured using a Bicinchoninic Acid Protein Assay kit (Beyotime, CA), and protein was denatured at 100 °C for 10 min with DualColor Protein Loading Buffer (Life, USA). Protein extracts were separated using 10% sodium dodecyl sulfate-polyacrylamide gel electrophoresis (SDS-PAGE). After electrophoresis, the proteins were transferred onto PVDF membranes (GE Healthcare Life Sciences, UK). Then membranes were blocked in 5% non-fat milk for 1 h, followed by incubation with primary antibodies overnight at 4 °C. After three washes with TBST, the blots were subsequently incubated with the corresponding secondary antibodies coupled to horseradish peroxidase (HRP) at room temperature for 2 h and developed using an electrochemiluminescence (ECL) western blot detection reagent (Thermo Scientific, USA). Antibody information is presented in Supplemental Table S2.

### Plasmid constructs, lentivirus Production, and cell transfection

Human NR4A3 cDNA (NM_173200.2) and CDKN2AIP cDNA (NM_017632.3) were subcloned into pEZ-Lv105 vector. The primers for cloning are provided in supplemental Table S3. And their shRNA lentiviral plasmids were supplied by GeneCopoeia (Guangzhou, China). Transfection of shRNAs, sgRNAs, oligonucleotides, and plasmids was performed using the Lipofectamine™ 2000 transfection reagent (Invitrogen, CA, USA) according to the manufacturer's instructions. The lentivirus was generated by co-transfecting 293T cells with a lentiviral vector and packaging plasmids. In the presence of 6 μg/mL polybrene (Sigma-Aldrich, St. Louis, MO, USA), HCC cells were infected with 1×10^6^ recombinant lentivirus-transducing units. All the shRNA and sgRNA target sequences are listed in supplemental Table S4.

### *In vitro* cell proliferation and colony formation assays

Cell proliferation was analyzed using the Cell Counting Kit-8 (CCK-8) and cell clone formation assays, according to the manufacturer's instructions. Briefly, 1×10^3^ cells were plated into 96-well plates per well and incubated at 37 °C for CCK-8 assay, and 10 μL CCK-8 solution was added into each well. After incubation for 2 h, the absorbance at A450 was measured using a microreader (Thermo Scientific, MA, USA). 1×10^3^) cells were plated in 6-well plates per well and incubated at 37 °C for colony formation assay for approximately 14 days. Colonies were fixed with 4% PBS-buffered formalin and stained with Giemsa for 2 h. Each experiment was performed in triplicate.

### Immunofluorescence imaging

Cells were washed three times with PBS in a confocal dish (NEST, 801001) and fixed with 4% paraformaldehyde for 15 min. Then permeabilized using 2% Triton X-100 PBS for 10 min with gentle shaking. The cells were stained with primary antibody overnight at 4 °C in a humidified chamber and stained with the corresponding secondary antibody conjugated to Alexa Fluor (Thermo Fisher Scientific) for 1 h after 3 times washed with PBS. Then incubated with 4', 6- diamidino-2-phenylindole (DAPI, Invitrogen, D1306) in a blocking solution for 30 min. Images were obtained with a Leica TCS SP8 confocal system (Leica, Microsystems).

### Flow cytometry analysis

Flow cytometry analysis was used to determine cell cycle distribution. Briefly, 1×10^6^ cells were plated in 6-well culture plates. The cells were treated with 2 mM thymidine (Sigma-Aldrich, USA) for 24 h to synchronize the cells at the G0/G1 phase. Then, cells were harvested using trypsin after releasing for 0 and 24 h, and washed with 1×PBS twice, fixed with 70% ethanol at -20 °C 16-18 h. Before flow cytometry analysis, cells were washed with 1×PBS buffer and resuspended with 400 mg/mL propidium iodide (PI), 10 mg/mL RNase (Sigma-Aldrich, USA), and 0.1% Triton X-100 in 200 µL 1×PBS on ice away from light for 30 min. The DNA content was quantified using Modfit 3.2 software.

### Tumor xenograft models

For the tumor xenograft assays, 2×10^6^ MHCC-LM3 or Li7 cells infected with NR4A3/vector, 2×10^6^ MHCC-97H, or MHCC-97L cells transfected with sgNC/sgNR4A3/shNC/shNR4A3-2/shNR4A3-3, 2×10^6^ MHCC-LM3 cells infected with Vector+NR4A3/NR4A3+shNC/NR4A3+shCDKN2AIP-1/NR4A3+shCDKN2AIP-2 were resuspended in 200 μL of serum-free DMEM and subcutaneously inoculated into one flank of each nude mouse (nu/nu, male, 4 weeks, n=6 or n=8 per group). After four weeks, all mice were sacrificed and the xenograft tumors were weighed. All the animal experimental protocols were approved by the Shanghai Medical Experimental Animal Care Commission.

### Chromatin immunoprecipitation (ChIP)

ChIP assay was performed according to the manufacturer's instructions (Millipore, Billerica, MD, USA). The MHCC-97L/HCC-LY10 cells were cross-linked with 10% formaldehyde and reversed with 1 M glycine. After washing with PBS, cells were harvested in Tissue Protein Extraction Reagent (Thermo Scientific, MA, USA) for 5 min on ice and centrifuged at 2,000 × *g* for 5 min. The precipitants were suspended in nuclei lysis buffer, and the DNA was crushed into fragments by sonication. Mouse anti-NR4A3 (Invitrogen, CA, USA) or mouse IgG with protein A/G-agarose beads (Sigma-Aldrich, USA) were added and incubated 16-18 h at 4 °C to immunoprecipitate DNA containing complexes. After washing, DNA was isolated and used for PCR analysis. Primers for the CDKN2AIP promoter were as follows: forward, 5′-tgtattttgttccagcatgcac-3′ and reverse, 5′-tgcatgaatcagaataagcaagc-3′.

### Luciferase assay

Recombinant plasmids with the normal and mutant promoter regions of NR4A3 were purchased from GeneCopoeia (Guangzhou, China). Cells were seeded into 96-well culture plates for 24 h and grown to approximately 90% confluence for 24 h. Then, the cells were co-transfected with the relevant reporter plasmids and the internal control PRL-TK reporter construct using Lipofectamine 2000 (Invitrogen, Madison, USA). After 48 h, firefly luciferase activity and Renilla luciferase activity were determined according to the manufacturer's instructions using a dual-luciferase reporter gene assay system (Promega, Madison, USA).

### Statistical analysis

Survival curves were plotted using the Kaplan-Meier method and compared using a log-rank test. The Student's t-test was used to test the differences between the two groups. p<0.05 was considered statistically significant (*p<0.05; **p<0.01).

## Supplementary Material

Supplementary figures and tables.

## Figures and Tables

**Table 1:** Correlation between NR4A3 levels in HCC patients and their clinicopathological characteristics

Clinicopathological feature	Number	Low expression N (%)	Medium expression N (%)	High expression N (%)	*p* Value
Gender					
Male	154	20 (12.99)	77 (50.00)	57 (37.01)	0.004*
Female	37	10 (27.03)	23 (62.16)	4 (10.81)	
Age					
≤50	131	21 (16.03)	68 (51.91)	42 (32.06)	0.977
>50	60	9 (15.00)	32 (53.33)	19 (31.67)	
HBsAg					
Absent	35	7 (20.00)	17 (48.57)	11 (31.43)	0.732
Present	156	23 (14.74)	83 (53.21)	50 (32.05)	
antiHBs					
Absent	183	30 (16.40)	97 (53.00)	56 (30.60)	0.128
Present	8	0 (0)	3 (37.50)	5 (62.50)	
HBeAg					
Absent	161	23 (14.29)	87 (54.04)	51 (31.68)	0.388
Present	30	7 (23.33)	13 (43.33)	10 (33.33)	
antiHBe					
Absent	109	20 (18.35)	60 (55.05)	29 (26.60)	0.154
Present	82	10 (12.20)	40 (48.78)	32 (39.02)	
antiHBc					
Absent	50	10 (20.00)	21 (42.00)	19 (38.00)	0.227
Present	141	20 (14.18)	79 (56.03)	42 (29.79)	
AFP (ng/mL)					
≤20	68	11 (16.18)	35 (51.47)	22 (31.35)	0.982
>20	123	19 (15.45)	65 (52.85)	39 (31.71)	
Tumor size (cm)					
≤5	98	9 (9.18)	56 (57.14)	33 (33.67)	0.038*
>5	93	21 (22.58)	44 (47.31)	28 (30.11)	
Histology grade					
I+ II	95	10 (10.53)	53 (55.79)	32 (33.68)	0.147
III+ IV	96	20 (20.83)	47 (48.96)	29 (30.21)	
Cirrhosis					
Absent	30	4 (13.33)	12 (40.00)	14 (46.67)	0.167
Present	161	26 (16.15)	88 (54.66)	47 (29.19)	
Intrahepatic metastasis					
Absent	128	21 (16.41)	62 (48.44)	45 (35.16)	0.284
Present	63	9 (14.29)	38 (60.32)	16 (25.40)	

*P* value represents the probability from a chi-square test for NR4A3 levels between variable subgroups. *P<0.05. Abbreviations: AFP, alpha-fetoprotein; HBV, hepatitis B virus.

## Abbreviations

NR4A3: Nuclear receptor subfamily 4 group A member 3
CDKN2AIP: CDKN2A interacting protein
HBV: hepatitis B virus
HCV: hepatitis C virus
TFs: Transcription factors
NOR1: neuron-derived orphan receptor 1
NBRE: NGFI-B response element-AAAGGTCA
ARF: alternative reading frame
IHC: immunohistochemistry
ChIP: chromatin immunoprecipitation
CCK8: Cell Counting Kit 8
HCC: hepatocellular carcinoma
IHC: immunochemistry
mRNA: messenger RNA
TCGA: The Cancer Genome Atlas
