# Supplementary material for: NR4A3 inhibits the tumor progression of hepatocellular carcinoma by inducing cell cycle G0/G1 phase arrest and upregulation of CDKN2AIP expression
Source: Int J Biol Sci. 2024 Oct 28;20(15):5850–67. doi: 10.7150/ijbs.95174 (PMC11628324; doi:10.7150/ijbs.95174)

Supplemental Information

NR4A3 inhibits the tumor progression of hepatocellular carcinoma by inducing cell cycle G0/G1 phase arrest and upregulation of CDKN2AIP expression

Xinge Zhao[1], Xuejie Min[1], Zhenyu Wang[2], Xiaoxia Chen[3], Chao Ge[1], Fangyu Zhao[1], Hua Tian[1], Taoyang Chen[4], Jinjun Li[1#]

[1] State Key Laboratory of Systems Medicine for Cancer, Shanghai Cancer Institute, Renji Hospital, Shanghai Jiao Tong University School of Medicine, Shanghai 200032, China

[2] Department of Oncology, Shanghai General Hospital, Shanghai Jiaotong University School of Medicine, Shanghai 200080, China

[3] School of Life Science and Technology, Shanghai Tech University, Shanghai 201210, China

[4] Department of Pathology, Qi Dong Liver Cancer Institute, Qidong 226220, China.

# Corresponding author

Supplemental Table S1 Primers used in this study

Primers for q-PCR	Sequences
GAPDH Forward	CTCCTCCTGTTCGACAGTCAGC
GAPDH Reverse	CCCAATACGACCAAATCCGTT
NR4A3 Forward	TGCGTCCAAGCCCAATATAGC
NR4A3 Reverse	GGTGTATTCCGAGCTGTATGTCT
CDKN2AIP Forward	CAAGATCACGCAAAAACCTCTG
CDKN2AIP Reverse	CGACCCTATACACGTTGAACTG
ADNP Forward	AGGCTGACAGTGTAGAGCAAG
ADNP Reverse	GACTGCCCCATTGAGTGATTTT
UBC Forward	GTGTCTAAGTTTCCCCTTTTAAGG
UBC Reverse	TTGGGAATGCAACAACTTTATTG
FOXA2 Forward	TGGAGCAGCTACTATGCAG
FOXA2 Reverse	CGTGTTCATGCCGTTCATC
PRNP Forward	CACGACTGCGTCAATATCACA
PRNP Reverse	CTCCATCATCTTAACGTCGGTC
VTN Forward	GCCTTCACCGACCTCAAGAAC
VTN Reverse	CCCCTGACAGTTGATGCGG
TCF7 Forward	TGCACATGCAGCTATACCCAG
TCF7 Reverse	TGGTGGATTCTTGGTGCTTTTC
TRIM32 Forward	TGTGGTTTGGTGTTATGTGAGC
TRIM32 Reverse	TAAGTTCCCGCAGACGAGTTA
BATF Forward	CGTATTGCCGCCCAGAAGAG
BATF Reverse	TCTGTTTCTCCAGGTCTTCGC

Supplemental Table S2 Antibody used in this study

Antibody	catalog	Dilution	Company
**For western blotting**			
NR4A3	MA5-26704	1:600	Invitrogen
CDKN2AIP	16615-1-AP	1:200	Proteintech
CDK4	12790S	1:1000	CST
CDK6	13331S	1:1000	CST
CyclinD1	2978S	1:1000	CST
PCNA	13110S	1:1000	CST
CAS9	Ab189380	1:1000	abcam
P53	P6749	1:1000	Sigma
P21	2947	1:1000	CST
γH2A.X	Ab81299	1:1000	abcam
β-actin	A3854	1:10000	Sigma
Secondary antibody	HRP conjugated goat anti-rabbit IgG	1:5000	Sigma
Secondary antibody	HRP conjugated goat anti-rabbit IgG	1:5000	Sigma
**For Immunohistochemistry**			
NR4A3	NBP2-46246	1:50	NOVUS
PCNA	13110S	1:3000	CST
Ki67	ab15580	1:500	abcam
Secondary antibody	Envision kit (HRP, mouse, DAB+)	Ready-to-use	DAKO

Supplemental Table S3 The primer sequences used for cloning in this study

NR4A3:	
Human-NR4A3 Forward	GCGGTAGGCGTGTACGGT
Human-NR4A3 Reverse	ATTGTGGATGAATACTGCC
CDKN2AIP:	
Human-CDKN2AIP Forward	GCGGTAGGCGTGTACGGT
Human-CDKN2AIP Reverse	ATTGTGGATGAATACTGCC

Supplemental Table S4 Target sequences used in this study

NR4A3 shRNAs:	
Human-NR4A3-sh#1	GCAGACATACAGCTCGGAATA
Human-NR4A3-sh#2	GCCTTCCTGCGTGTACCAAAT
Human-NR4A3-sh#3	GCCCTTGTCCGAGCTTTAACA
Human-NR4A3-sh#4	GCTTTCAAATGAATGATCAGG
Control shRNA:	
Control shRNA	ACAGAAGCGATTGTTGATC
NR4A3 sgRNA:	
Human-NR4A3-sg	caccgAGTCTGTTAAAGCTCGGACAgttt
Human-NC-sg	GTATTACTGATATTGGTGGG
CDKN2AIP shRNAs:	
Human-CDKN2AIP-sh#1	GTGACAGATGCTCCAACCTAT
Human-CDKN2AIP-sh#2	GTTCTGCAGTTGAGCAAGATC
Human-CDKN2AIP-sh#3	GTCAGAATAGCTCTACAAGTG
Human-CDKN2AIP-sh#4	GTTGCCAAGAAGGGGTAGAAG
Control shRNA:	
Control shRNA	ACAGAAGCGATTGTTGATC
CDKN2AIP sgRNA:	
Human-CDKN2AIP-sg	caccgTCGGGTCCCGCTCTCGGCGTgttt
Human-NC-sg	GTATTACTGATATTGGTGGG

Supplementary Figure 1. NR4A3 is a potential biomarker in HCC. (A) The expression of NR4A3 in different cancers of TCGA dataset was analyzed by the TCGA database. (B) The expression of NR4A3 in HCC tissues compared with adjacent normal tissues was analyzed using datasets from TCGA. (C) The correlation between NR4A3 expression and tumor stage in HCC was analyzed using TCGA datasets. (D) Waterfall plot showing the protein level of NR4A3 in HCC compared with adjacent noncancerous tissues from 28 patients determined using western blot. (Red histogram: NR4A3 overexpressed more than two times. Blue histogram: NR4A3 decreased more than two times). **, p<0.01.

Supplementary Figure 2. NR4A3 is efficiently up-regulated in mRNA levels *in vitro* and *in vivo* in HCC. (A) q-PCR analysis of NR4A3 expression in HCC cell lines and immortalized normal hepatocyte MIHA. (B) Western blot analysis of NR4A3 expression in HCC cell lines and immortalized normal hepatocyte MIHA. The mRNA expression of NR4A3 following overexpression (C) or knockdown (D) in HCC cells was determined by qPCR. The mRNA expression of NR4A3 in tumor tissues collected from nude mice with tumor xenografts inoculated with NR4A3-overexpressing (E-F) or knockout(G)/knockdown (H) cells was determined by qPCR. For qPCR, values were normalized to GAPDH.

Supplementary Figure 3. NR4A3 inhibits HCC cell proliferation. (A) Colony formation assay showed that overexpression of NR4A3 inhibited the proliferation of HCC cells. (B) Colony formation assay showed that down-regulation of NR4A3 promoted the proliferation of HCC cells. (C-D) CCK8 assay revealed that the proliferation of HCC cells was promoted by knockout of NR4A3. (E-F) Knockout of NR4A3 accelerated cell cycle progression. (G-H) Knockout of NR4A3 promoted the colony formation ability of HCC cells. *, p<0.05; **, p<0.01.

Supplementary Figure 4. NR4A3 suppressed HCC progression by inducing CDKN2AIP expression. Binding of NR4A3 to the Skp2 (A-B) promoter was performed by ChIP using the antibody against NR4A3 and negative control (IgG) in MHCC-97H and MHCC-97L cells. Binding of NR4A3 to the Skp2 (C-D) or CDKN2AIP (E-F) promoter was performed by ChIP using the antibody against NR4A3 and negative control (IgG) in MHCC-97H-sgNR4A3 and MHCC-97L-sgNR4A3 cells. *, p<0.05; **, p<0.01.

Supplementary Figure 5. Overexpression of CDKN2AIP inhibits HCC cell proliferation. (A) The panel showed the overlap up-regulated genes examined using q-PCR after over-expressing of NR4A3 in MHCC-LM3 cells. (B) mRNA of CDKN2AIP was up-regulated in HCC NR4A3 over-expressing cells. (C) mRNA of CDKN2AIP was down-regulated in HCC shNR4A3 cells. The mRNA expression of NR4A3 (D) and CDKN2AIP (E) following knockout of NR4A3 in HCC cells was determined by qPCR. The mRNA expression of NR4A3 (F) and CDKN2AIP (G) following knockout of CDKN2AIP after overexpressing of NR4A3 in HCC cells was determined by qPCR. (H-J) CDKN2AIP inhibited the proliferation of HCC cells by CCK8 assay. (K-L) Overexpression of CDKN2AIP inhibited the colony formation ability of HCC cells.

Supplementary Figure 6. NR4A3 inhibits cell proliferation by up-regulating CDKN2AIP expression. (A) Knockout of CDKN2AIP reversed the inhibitory effect of NR4A3 on cell

proliferation using CCK-8 assay. (B) Knockout of CDKN2AIP reversed the inhibitory effect of NR4A3 on cell cycle distribution. (C-D) Knockout of CDKN2AIP reversed the inhibitory effect of NR4A3 on cell colony formation. (E) Western blot analysis of cell cycle associated proteins in the G1 phase (CDK6, CDK4, CyclinD1) and proliferation marker (PCNA) expression in NR4A3 knockdown cells. (F) Knockdown of CDKN2AIP reversed the inhibitory effect of NR4A3 on cell colony formation. (G) The NR4A3 and CDKN2AIP protein levels were detected by Western blot in xenografts. Tumor tissues collected from nude mice with tumor xenografts derived from MHCC-LM3 cell with silencing CDKN2AIP under NR4A3-overexpressed condition. β-actin was used as a loading control. The bar graph showed quantitative analysis data with three replicates. *, $p<0.05$; **, $p<0.01$.

Supplementary Figure 7. Clinical correlation between CDKN2AIP and NR4A3. (A) The protein levels of NR4A3 in 42 paired HCC (T) and adjacent normal (N) samples (15-42) were measured using western blot. β-actin was used as a loading control.

Supplement Figure 1

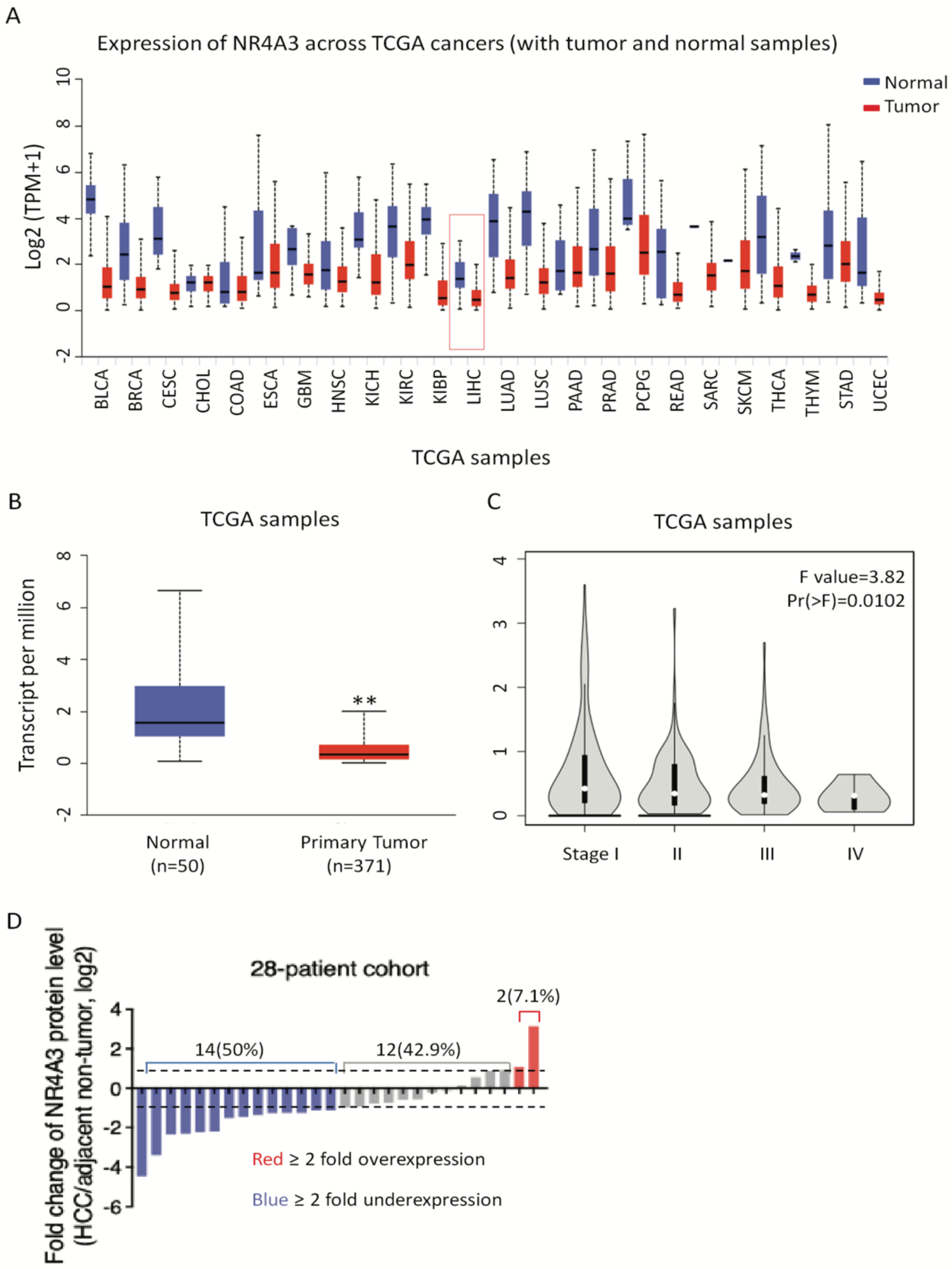

Supplement Figure 2

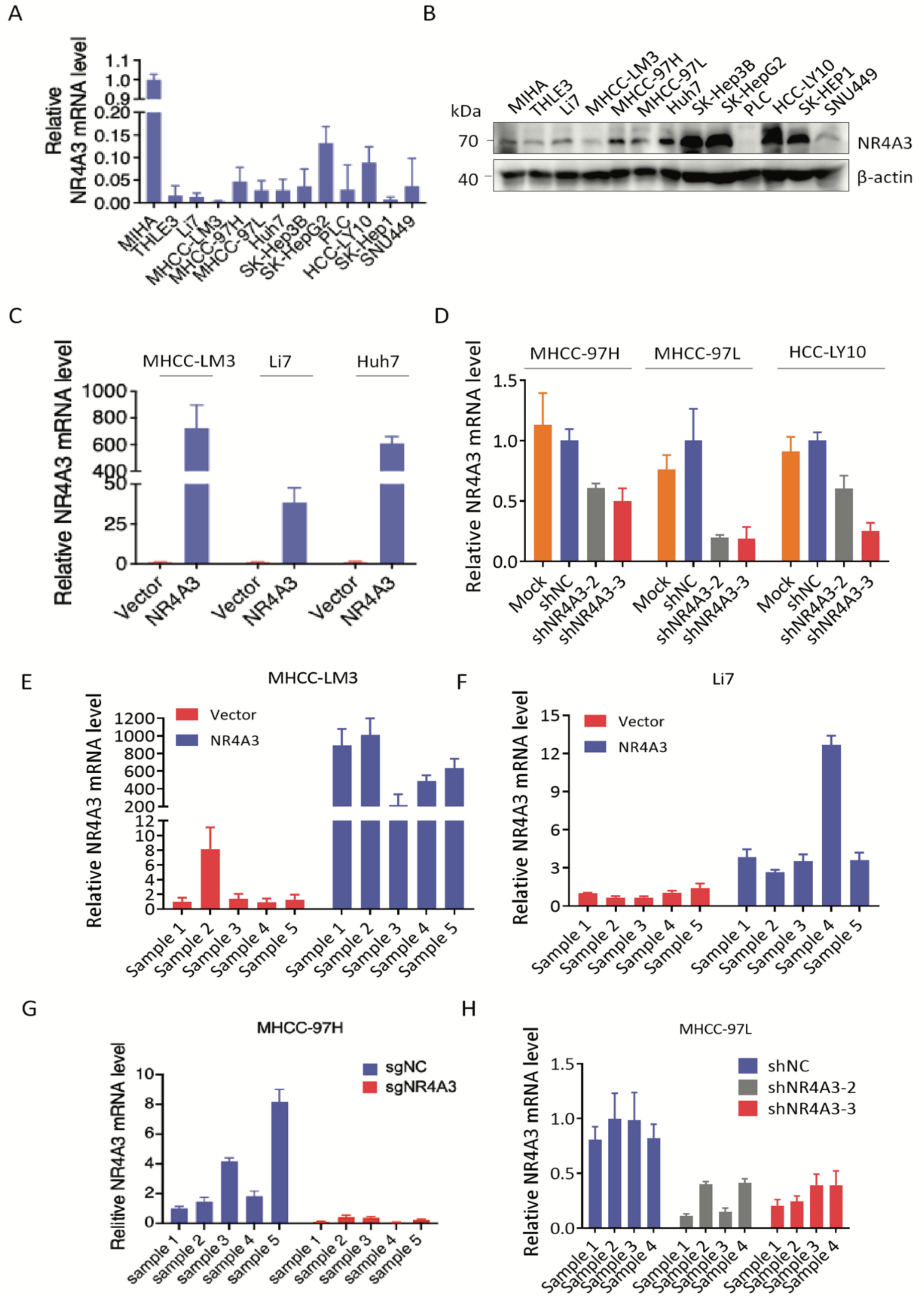

Supplement Figure 3

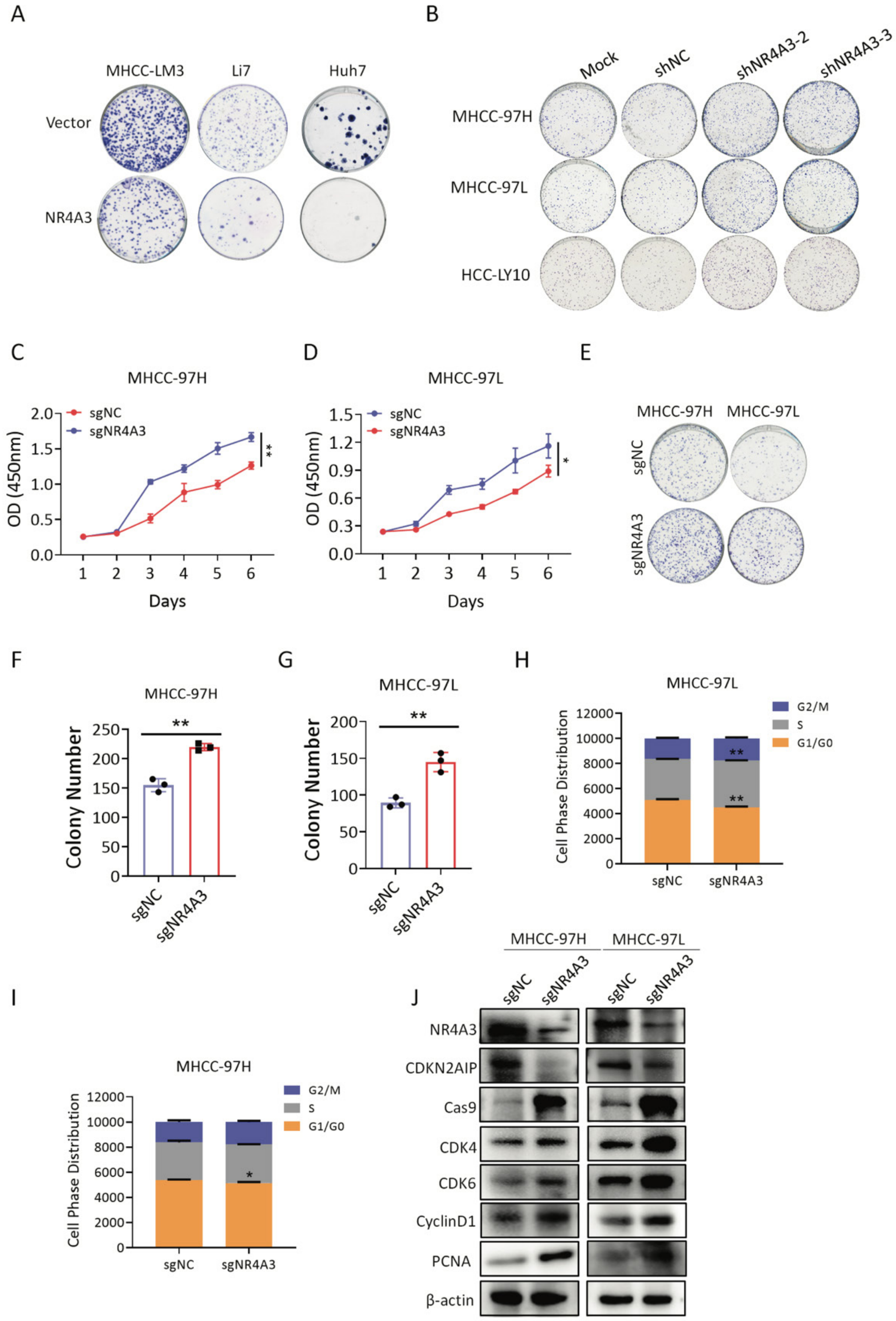

Supplement Figure 4

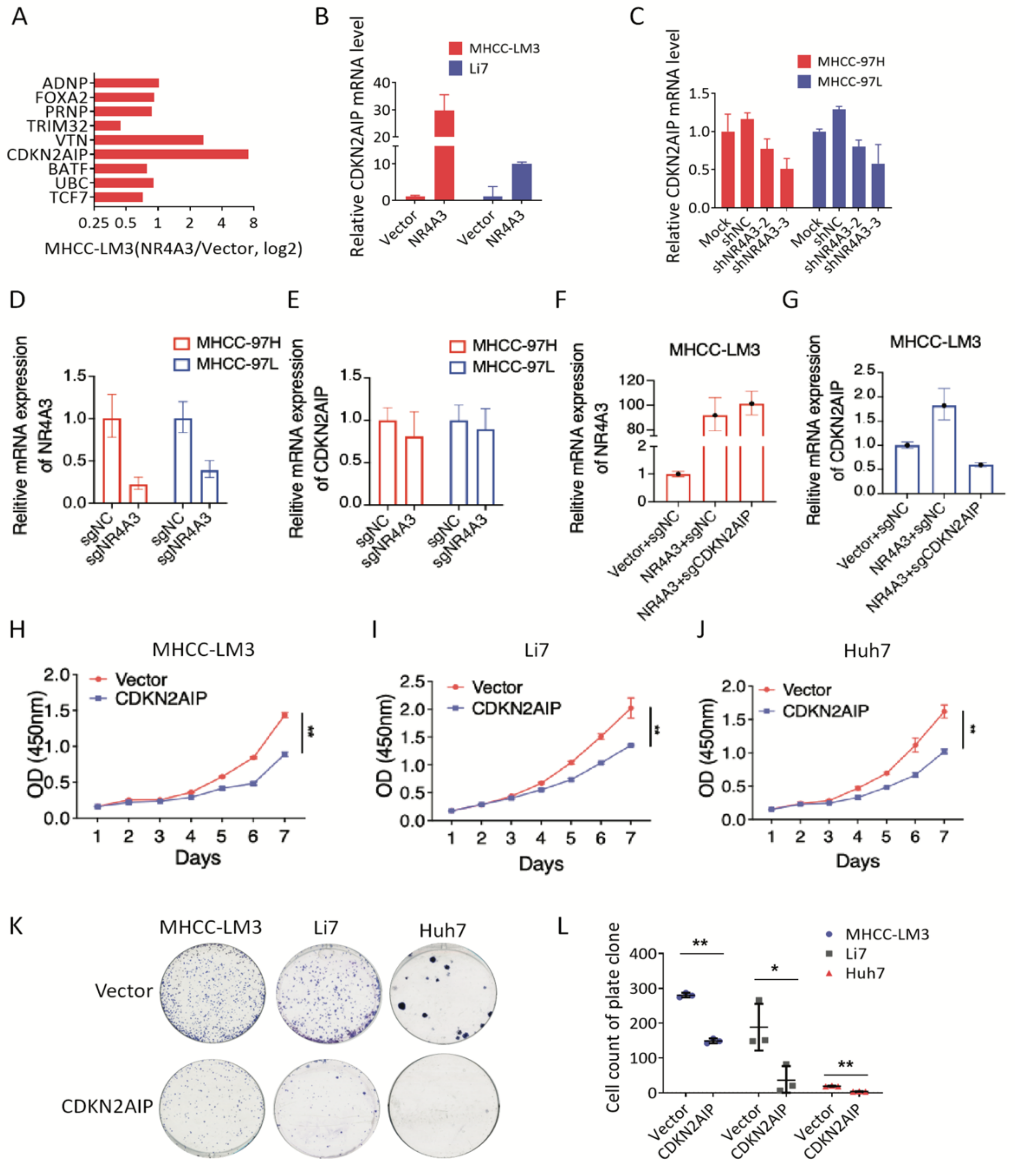

Supplement Figure 5

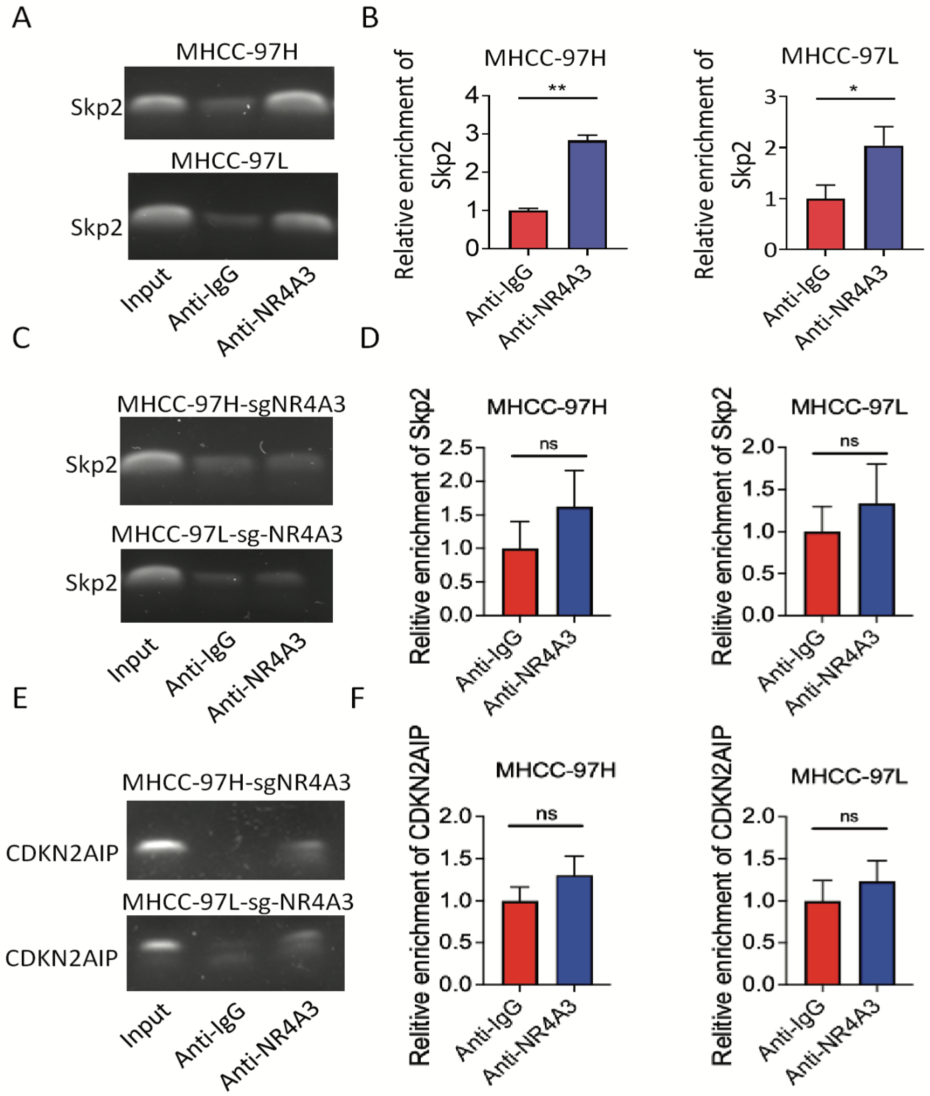

Supplement Figure 6

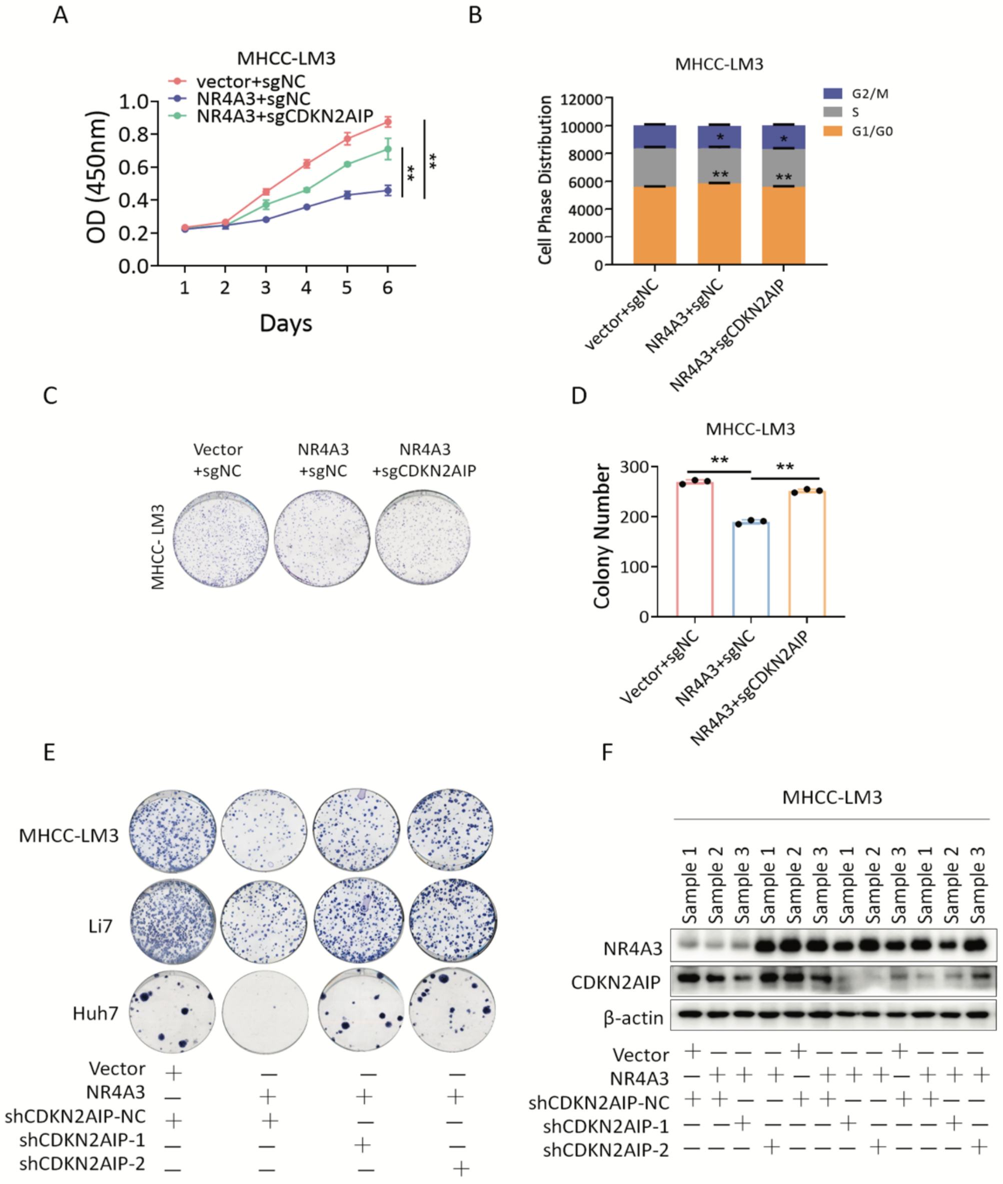

Supplement Figure 7

A

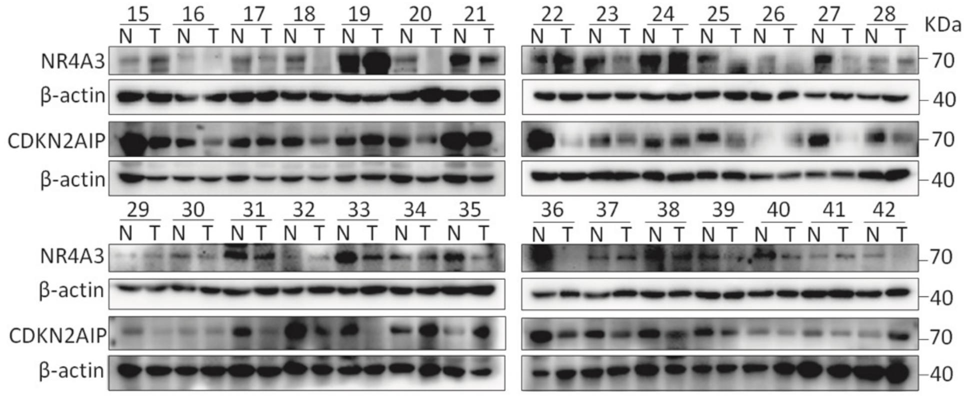

Supplement: Supplementary file 1 — Supplementary figures and tables. [file ijbsv20p5850s1.pdf]
